# Artificial Q‐Grader: Machine Learning‐Enabled Intelligent Olfactory and Gustatory Sensing System

**DOI:** 10.1002/advs.202308976

**Published:** 2024-04-06

**Authors:** Moonjeong Jang, Garam Bae, Yeong Min Kwon, Jae Hee Cho, Do Hyung Lee, Saewon Kang, Soonmin Yim, Sung Myung, Jongsun Lim, Sun Sook Lee, Wooseok Song, Ki‐Seok An

**Affiliations:** ^1^ Thin Film Materials Research Center Korea Research Institute of Chemical Technology Daejeon 34114 Republic of Korea; ^2^ National Nano Fab Center (NNFC) Daejeon 34141 Republic of Korea; ^3^ Department of Medical Artificial Intelligence Konyang University Daejeon 35365 Republic of Korea; ^4^ School of Electronic and Electrical Engineering Sunkyunkwan University Suwon 16419 Republic of Korea

**Keywords:** gas sensor, liquid sensor, machine learning, surface engineering, zinc oxide

## Abstract

Portable and personalized artificial intelligence (AI)‐driven sensors mimicking human olfactory and gustatory systems have immense potential for the large‐scale deployment and autonomous monitoring systems of Internet of Things (IoT) devices. In this study, an artificial Q‐grader comprising surface‐engineered zinc oxide (ZnO) thin films is developed as the artificial nose, tongue, and AI‐based statistical data analysis as the artificial brain for identifying both aroma and flavor chemicals in coffee beans. A poly(vinylidene fluoride‐co‐hexafluoropropylene)/ZnO thin film transistor (TFT)‐based liquid sensor is the artificial tongue, and an Au, Ag, or Pd nanoparticles/ZnO nanohybrid gas sensor is the artificial nose. In order to classify the flavor of coffee beans (acetic acid (sourness), ethyl butyrate and 2‐furanmethanol (sweetness), caffeine (bitterness)) and the origin of coffee beans (Papua New Guinea, Brazil, Ethiopia, and Colombia‐decaffeine), rational combination of TFT transfer and dynamic response curves capture the liquids and gases‐dependent electrical transport behavior and principal component analysis (PCA)‐assisted machine learning (ML) is implemented. A PCA‐assisted ML model distinguished the four target flavors with >92% prediction accuracy. ML‐based regression model predicts the flavor chemical concentrations with >99% accuracy. Also, the classification model successfully distinguished four different types of coffee‐bean with 100% accuracy.

## Graphical abstract

In this study, AI‐driven sensors using surface‐engineered ZnO thin films are developed and designed to mimic human olfactory and taste systems. Employing machine learning models, the system achieves over 92% accuracy in identifying coffee bean flavors and 99% in predicting chemical concentrations. This sensor system constitutes a promising candidate for IoT applications in environmental monitoring and early disease diagnosis.

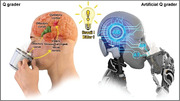

## 1 Introduction

Exploitation of portable sensors accompanied with artificial intelligence (AI) system for sensing gas and liquid phase substances, that mimic the olfactory and gustatory systems of humans, serve as a stimulus to buttress the large‐scale deployment of the newly encountered Internet of Things (IoT) devices.^[^ ^1^, ^2^, ^3^, ^4^ ^]^ Judicious combination of artificial olfactory and gustatory sensors renders it potentially suitable for multifaceted IoT sensor applications in monitoring air, water, and food quality, as well as breath‐ and saliva‐based early disease diagnosis.^[^ ^5^, ^6^, ^7^, ^8^, ^9^, ^10^ ^]^ For this purpose, the innovative exploitation of AI Q‐grader can be an applicable and unprecedented model system with which to harness the capability of personalized artificial olfactory and gustatory functions. A certified Q‐grader is a skilled professional who evaluates the quality of coffee, that is, the flavor (sweetness, bitterness, and sourness), appearance, aroma, and conditions of raw coffee beans. Thus far, manifold approaches to discern the aroma and flavor of coffee have been fulfilled via gas chromatography‐mass spectroscopy (GC‐MS), Raman spectroscopy, NIR spectroscopy, and cyclic voltammetry, as well as commercially available metal oxide‐based sensors, electrochemical gas sensors, and lipid/polymer membrane‐type liquid sensors.^[^ ^11^, ^12^, ^13^, ^14^, ^15^, ^16^, ^17^, ^18^, ^19^ ^]^ Although progress has been made, the accomplishment of AI Q‐grader remains a challenging task evidently and is far from the above‐mentioned idealistic scenario because of strict restrictions on dual detection for gas and liquid analytes, attainment of portable and inexpensive sensors, and dual classification of aroma and flavor of coffee enabled by conjugating machine learning (ML). Here, we unprecedentedly and strategically designed a self‐discriminable AI Q‐grader with olfactory and gustatory sensors based on surface‐engineered zinc oxide (ZnO) with target functionalities combined with an AI‐based statistical data analysis. ZnO, an archetypical metal oxide, constitutes an impeccable candidate for conjugating semiconductor‐type gas and liquid sensor applications as it can detect diverse analytes owing to its inherent physical properties, is inexpensive to synthesize, and is environmentally friendly.^[^ ^20^, ^21^, ^22^, ^23^, ^24^ ^]^ To authorize the dual functionalities pertaining to analyte selectivity of single constituent ZnO for olfactory and gustatory sensors, we primarily aim to establish well‐designed and adaptive surface engineering induced by organic‐inorganic hybridization (polymer‐metal oxide) and metal‐semiconductor hybridization (metal nanoparticles‐metal oxide). In our previous works, the adaptive surface engineering of metal oxides enables to realize newly synergistic properties, providing unprecedented possibilities for next‐generation sensors based on hybrid nanomaterials.^[^ ^25^, ^26^, ^27^ ^]^ In addition, to categorize the origin and flavor of coffee beans, we innovatively established a ML model for gas and liquid classification and concentration prediction onto a 2D principal component (PC) space, enabling high prediction accuracy and readability based on pre‐manipulated feature data from PC analysis (PCA).^[^ ^28^, ^29^ ^]^

## 2 Results and Discussion

**Figure 1:**
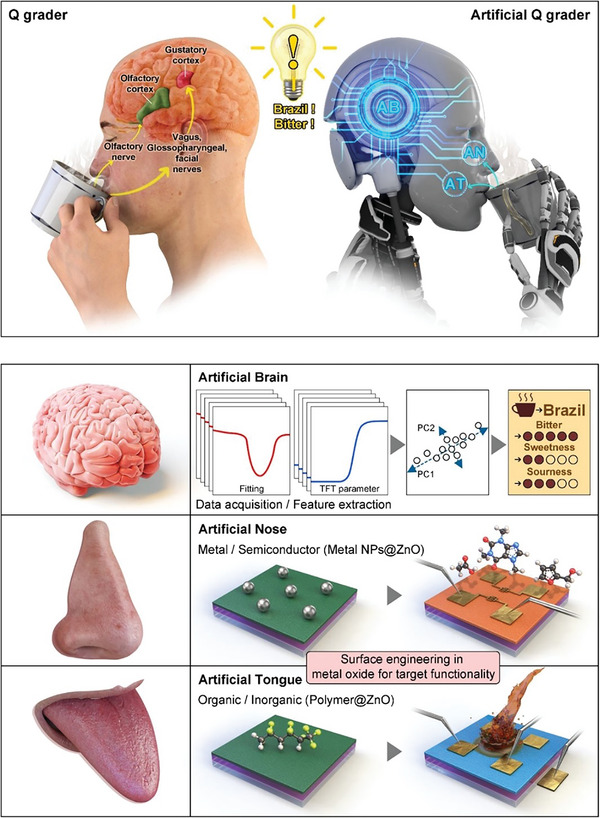
Conceptual representation of artificial Q‐graders resembling human olfactory and gustatory systems for identifying the aroma and flavor of coffee. The self‐discriminable artificial Q‐graders are attained by rationally coordinating artificial brain (AB), artificial nose (AN), and artificial tongue (AT). The ML‐based artificial brain involves sensor data acquisition, feature extraction, and PCA‐assisted classification of diverse gases and liquids extracted from coffee. The artificial nose is a metal/semiconductor (Au, Ag, and Pd NPs@ZnO) hybrids‐based two‐terminal interdigitated gas sensor that can discern four different coffee beans (Papua New Guinea, Brazil, Ethiopia, and Colombia‐decaffeine). The artificial tongue is an organic/inorganic (PVDF@ZnO) hybrid‐based thin film transistors (TFTs) type liquid sensor to discriminate sweet (ethyl butyrate, 2‐furanmethanol), sour (acetic acid), and bitter (caffeine) flavors.

**Figure** **1** depicts the pictorial representation of artificial Q‐graders for identifying the aroma and flavor of coffee by mimicking the biological olfactory and gustatory system in humans. In the human olfactory and gustatory systems, odor and flavor identification through preprocessed electrical signals triggered by chemical reactions between receptor cells and gases or liquids is proceeded by following neural pathways: i) Aroma from coffee – Nose – Olfactory nerve – Olfactory cortex – Odor identification, ii) Flavor from coffee – Tongue – Vagus nerve/Glossopharyngeal nerve/Facial nerve – Gustatory cortex – Flavor identification, as illustrated in Figure 1. Here, self‐discriminable artificial Q‐graders were rationally devised by coordinating artificial brain enabled by ML, artificial nose, and artificial tongue based on surface‐engineered nanohybrids with target functionalities. The sensor data acquisition relevant to electrical responses corresponding to aroma and flavor of coffee was implemented using an artificial nose and artificial tongue. In order to classify the origin (Papua New Guinea, Brazil, Ethiopia, and Colombia‐decaffeine) and flavor (sweet, sour, and bitter) of coffee beans, PCA‐assisted classification was performed in the artificial brain based on features extracted from the response curves. To give the dual functionalities to facilitate artificial nose and artificial tongue using a single constituent ZnO, adaptive surface engineering stimulated by synergistic coupling of metal‐semiconductor (Au, Ag, and Pd nanoparticles (NPs)‐ZnO thin film) and organic‐inorganic materials (poly(vinylidene fluoride‐co‐hexafluoropropylene) (PVDF‐HFP)‐ZnO thin film) was both adopted. These viable and succinct strategies contribute to outperformed analyte sensitivity as well as selectivity through the spillover effects of metal NPs on ZnO and the strong dipole moment of PVDF‐HFP on ZnO.^[^ ^25^, ^27^ ^]^

**Figure 2:**
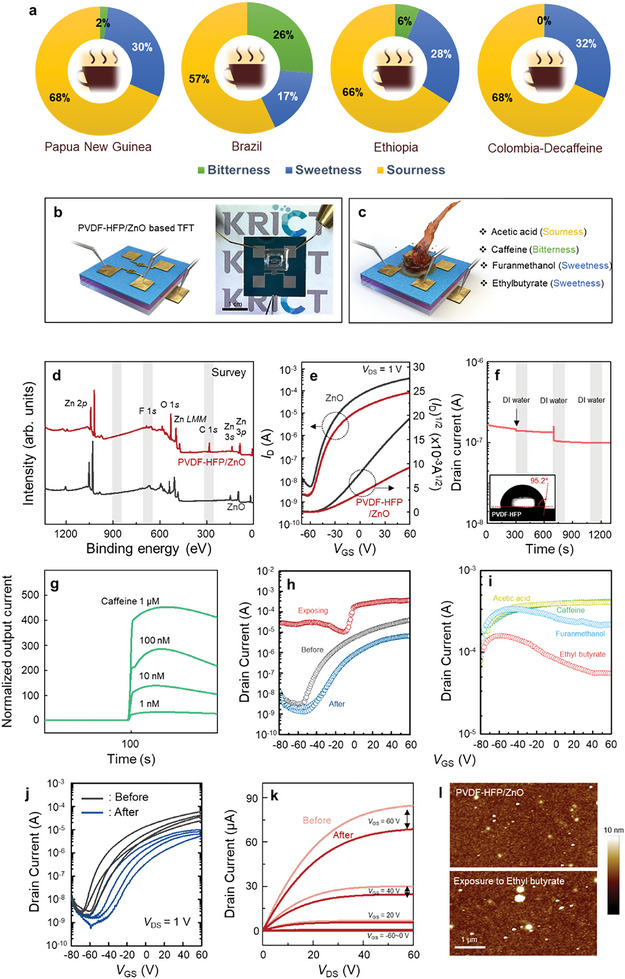
a) Proportions of the chemicals responsible for bitterness, sweetness, and sourness in the four different coffee beans, as evidenced by GC‐MS analysis. b) Schematic depiction coupled with optical microscope images of the device structure of the PVDF‐HFP/ZnO TFTs‐type liquid sensor. c) Selected chemicals (acetic acid, caffeine, furanmethanol, ethyl butyrate) for identifying the flavor of four coffee beans. d) Representative XPS survey spectra collected from ZnO and PVDF‐HFP/ZnO thin films. e) Transfer characteristics of ZnO TFTs with and without PVDF‐HFP. f) DI water responsitivities of liquid sensors based on surface‐engineered ZnO with PVDF‐HFP; inset: photograph of the contact angle for a water drop on the PVDF‐HFP/ZnO surface. g) Real‐time responses of the surface‐engineered TFT‐based sensor toward various concentrations of caffeine (bitterness) under typical operating conditions (*V*_DS_ = 1 V and *V*_GS_ = 5 V). Transfer characteristics of surface‐engineered TFT‐based sensors h) before and after exposure, and exposing to the caffeine solution and i) exposing to chemical (acetic acid, caffeine, furanmethanol, and ethyl butyrate). j) Transfer and k) output characteristics of the sensors before and after exposure to ethyl butyrate. l) Representative AFM height profiles of ZnO with PVDF‐HFP before and after ethyl butyrate sensing.

As a proof‐of‐principle system, we analyzed the volatile chemicals in the coffee beans of four different origins (Papua New Guinea, Brazil, Ethiopia, and Colombia‐decaffeine) via GC‐MS analysis, as represented in **Figures** **2**a and S1 (Supporting Information). Detailed measurement conditions are summarized in Table S1 (Supporting Information). Formic acid and acetic acid (sourness), (furan‐2‐yl)methanol, 2‐furancarboxaldehyde, 2‐furanmethanol, furan (sweetness), and caffeine (bitterness) are explicitly discernible, irrespective of the origin of coffee beans. The proportion of bitter, sweet, and sour chemicals in the coffee beans from Papua New Guinea were 2%, 30%, and 68%; from Brazil, 26%, 17%, and 57%; from Ethiopia, 6%, 28%, and 66%; and from Colombia‐decaffeine, 0%, 32%, and 68%, respectively, which hints a discriminable correlation between the origin of beans and flavor of coffee. To accomplish highly sensitive detection of volatile chemicals in coffee beans, we fabricated a bottom‐gate/top‐contact TFT‐based sensor based on PVDF‐HFP/ZnO by adaptive surface engineering, as represented in Figure 2b. We prudently targeted representative chemicals (acetic acid, caffeine, 2‐furanmethanol, ethyl butyrate) for identifying the coffee flavor, as displayed in Figure 2c. Figure S2 (Supporting Information) shows the Fourier transform infrared (FTIR)‐attenuated total reflectance (ATR) spectra for aqueous solutions of caffeine, acetic acid, 2‐furanmethanol, and ethyl butyrate. A strong absorption band was observed at 1757 cm^−1^, corresponding to the symmetrical stretching of an ester carbonyl (─C═O) in all four targets. In addition, a band was observed at 1080 cm^−1^ corresponding to ─C─O─ stretching vibrations in the ─O─C═O group. The results of chemicals that affect coffee flavor are encompassed in Table S2 (Supporting Information).^[^ ^30^, ^31^, ^32^, ^33^, ^34^ ^]^ The data gained from FTIR‐ATR spectrum supports the core idea that the structural states of targeted chemicals are sensitive to interact with the C─F dipoles of PVDF‐HFP. The functional groups of 0.05 wt.% PVDF‐HFP dissolved in *N*,*N*‐dimethylformamide (DMF) were established by FTIR in the ATR mode, as represented in Figure S3 (Supporting Information). Three strong peaks were observed at 1670.73, 1390.90, and 1094.62 cm^−1^ corresponding to the C═O stretching, ─CH_3_ bending, and C─N stretching vibrations, respectively, of DMF.^[^ ^35^ ^]^ The peak at 1201.61 cm^−1^ corresponds to the ─CF_2_ symmetric stretching vibration of the PVDF‐HFP polymer, whereas that at 880.63 cm^−1^ is attributed to the adsorption of the C─F band in the amorphous phase by the additional HFP.^[^ ^36^, ^37^ ^]^ X‐ray photoelectron spectroscopy (XPS) analysis was conducted to identify the chemical states of the PVDF‐HFP/ZnO nanohybrids. As displayed in Figure 2d, the XPS survey spectra exhibit all constituent chemical compositions within the sample surface such as Zn, O, C, and F. The chemical states with binding energy (*E*_B_) = 529.87 and 531.66 eV in the O 1*s* core level spectra correlated with the ZnO crystal lattice and oxygen vacancies are simultaneously discernible in both PVDF‐HFP/ZnO and ZnO films. In the Zn 2*p* core level spectrum of pristine ZnO, the doublet peaks associated with Zn 2*p_3/2_* and Zn 2*p_1/2_* bonding states are detected at *E*_B_ = 1021.04 and 1044.14 eV, respectively.^[^ ^38^ ^]^ After surface engineering of the ZnO with PVDF‐HFP, the peak intensities are drastically attenuated compared to those of the pristine ZnO. The PVDF‐HFP polymer layer containing ─CF_2_─CH_2_─, ─CF_2_, and ─CH_2_, mainly contributed to the F 1*s* and C 1s core level spectra. The prominent peak at 687.53 eV in the F 1s core level spectrum stems from ─CF_2_─CH_2_─ and the resultant bonding states of ─CH_2_ and ─CF_2_ were observed at 285.52 and 289.92 eV in the C 1s core level spectra (Figure S4, Supporting Information)^[^ ^39^, ^40^, ^41^ ^]^. The transfer characteristics of the ZnO‐based TFTs with and without PVDF‐HFP were compared in the saturation regime, as displayed in Figure 2e. The ZnO‐based and PVDF‐HFP/ZnO‐based TFTs demonstrated an average field‐effect mobility of 0.65±0.55 and 0.21±0.04 cm^2^ V^−1^ s^−1^ with an *I*_on_/*I*_off_ of >10^5^ and >10^4^, respectively. The reduced electrical transport in PVDF‐HFP/ZnO TFT may arise from the electron‐withdrawing effect of C─F in PVDF‐HFP, which decreases the concentration of electrons in *n*‐channel devices, as well as the presence of trapped impurities in PVDF‐HFP during its solution process. Despite the degradation in the carrier mobility of the prepared TFTs, they entail adequate device performance suitable for sensing analytes in the aqueous phase (Figure S5 and Table S3, Supporting Information). Here, we demonstrate an amperometric flavor sensor based on PVDF‐HFP/ZnO‐based TFTs toward chemical analytes involving caffeine (bitterness), acetic acid (sourness), 2‐furanmethanol and ethyl butyrate (sweetness), as ascertained by GC‐MS analysis of four different origins (Papua New Guinea, Brazil, Ethiopia, and Colombia‐decaffeine) of coffee beans. To secure the reliability of the water‐resistant liquid‐phase sensing with PVDF‐HFP, we first measured deionized (DI) water responsivities of hydrophobic surface‐modified PVDF‐HFP/ZnO‐based sensors. Continuous exposure to DI water did not produce signals, as indicated in Figure 2f. Prior to detecting the chemicals, the baseline current was estimated using DI water. After stabilizing the output current, the real‐time and transfer sensing characteristics of the TFT‐based sensors were measured under ambient conditions (e.g., room temperature and atmospheric pressure in the presence of humidity at 25%) using a semiconductor parameter analyzer (Keithley 4200‐SCS). As illustrated in Figure 2b,c, a sensing platform was prepared by placing a polydimethylsiloxane mold reservoir on the TFT sensor, and *V*_D_ and *V*_G_ were set to 1 and 5 V, respectively. The output current responses increased with increasing caffeine concentration, and the detection limit was as low as the nanomolar range, as shown in Figure 2g. The sensitivity (i.e., normalized drain current) gained from the sensors was calculated by dividing the measured data by the baseline current. For *n*‐channel ZnO‐based sensors with the PVDF‐HFP layer, electron transport is activated under ion‐dipole interacting with the flavor chemicals containing electron‐donating groups, which releases the trapped carrier in ZnO channel region after PVDF‐HFP functionalization. Thus, the drain current increased after analyte injection, indicating positive sensing behavior. Infinitesimal quantities of target analytes can be discerned with accuracy and precision via TFT‐type sensing platforms by monitoring the following parameters: on‐state current (*I*_on_), off‐state current (*I*_off_), on‐off current ratio (*I*_on_/*I*_off_), subthreshold swing value (*S*.*S*.), threshold voltage (*V*_th_), and field‐effect mobility (*μ*). Figure 2h,i displays the evolution of transfer curves extracted from the PVDF‐HFP/ZnO‐based sensor which was exposed to a caffeine solution and flavor chemicals (acetic acid, caffeine, 2‐furanmethanol, and ethyl butyrate) under three different stages; initial state (before exposure), under exposure, and after cleaned by DI water (after exposure). Exposure to an aqueous caffeine solution resulted in a positive shift in the threshold voltage and an increase in both the on‐ and off‐currents. Figure S6 (Supporting Information) shows the transfer characteristics of the sensors for acetic acid, 2‐furanmethanol, and ethyl butyrate solution. Figure 2j,k shows the representative transfer and output characteristics of the TFT‐type sensors before and after the ethyl butyrate detection. Despite the slight change in the drain current of four TFT‐based sensors, all these sensors exhibited ample device performance for sensing analytes in the aqueous phase. In addition to high stability, each sensor current in the transfer curves provides a low device‐to‐device variation. It is noted that these results hint successful implementation of TFTs as reliable chemical sensors ascertained by good reproducibility and stable sensing in aqueous media. It is noted that these results hint successful implementation of TFTs as reliable chemical sensors is ascertained by good repeatable and stable sensing in aqueous media. Figure 2l corroborates a morphological analysis of the TFT‐type sensor before and after ethyl butyrate sensing ascertained using atomic force microscopy (AFM) in height mode. The PVDF‐HFP molecules aggregated on the surface of the ZnO thin films after the sensing trials, likely owing to the binding interactions between the PVDF polymer and ethyl butyrate.

**Figure 3:**
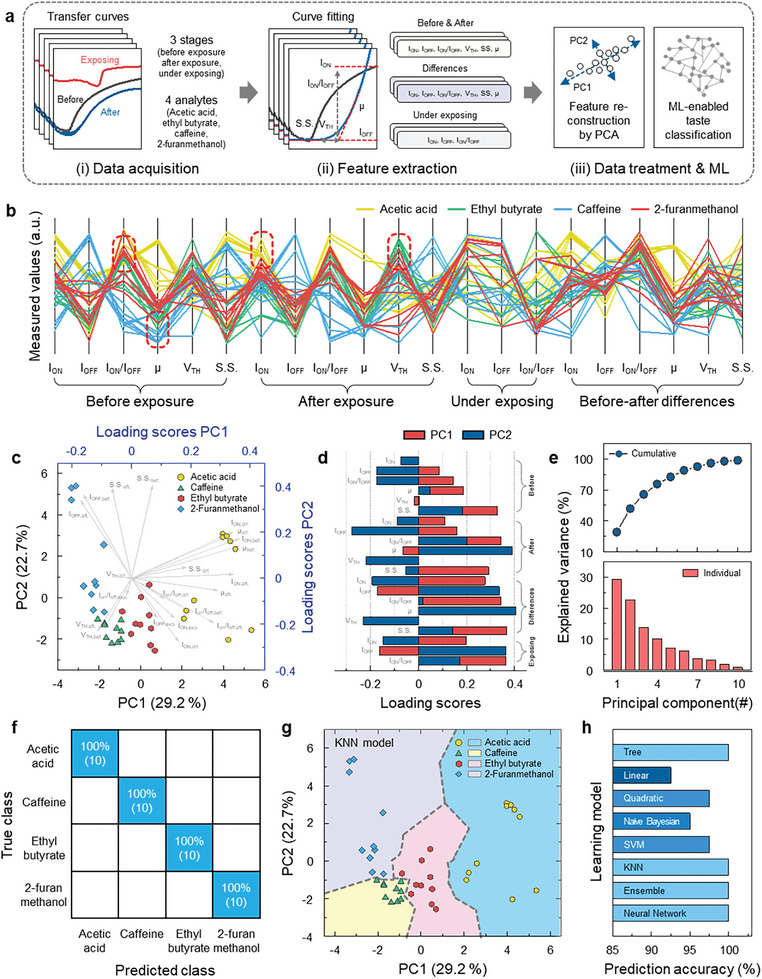
Machine‐learning assisted‐predictive artificial tongue. a) Schematic illustration of the data preparation for PCA‐assisted classification of four different chemicals (acetic acid, ethyl butyrate, caffeine, and 2‐furanmethanol) associated with the flavor of coffee. b) Parallel coordinate plot of 21 extracted parameters from the transfer‐curve measurements of surface‐engineered nanohybrid TFTs under three different stages: i) before exposing the analytes, ii) after exposing analytes, and iii) under exposing analytes, and iv) parameter changes between the before and after exposure c) PCA plot for the four different type of flavor chemicals (acetic acid, ethyl butyrate, caffeine, and 2‐furanmethanol). d) Loading score of two representative principal components (PC1 and PC2) for the 21 extracted parameters. e) Explained variance plot by principal components. PCA‐assisted ML results. f) Confusion matrix of the four different flavor analytes from the k‐nearest neighbor (kNN) trained model. g) Decision map of the four different flavor analytes classified by a kNN ML model with *k*‐fold validation (*k* = 5). h) Summary of the prediction accuracy of varied training models.

**Figure** **3**a displays a schematic illustration for general process to manifest the PCA‐driven recognition of four different flavor chemicals: acetic acid (sourness), ethyl butyrate (sweetness), 2‐furanmethanol (sweetness), and caffeine (bitterness). First, we collected the transfer curves of surface‐engineered nanohybrid TFTs for the flavor chemicals at three different stages: before, under, and after exposure to the analytes, as seen in Figure 2. The transfer curve corroborates the flavor‐chemical‐dependent dynamics in the electrical transport behavior. Specifically, the analytes‐TFT dynamics can be represented as curve fitting and performance parameters extracted from the transfer curve, that is *I*_on_, *I*_off_, *I*_on_/*I*_off_, *S*.*S*., *V*_th_, and *μ*. Since two classes of datasets consisting of labeled data (or answer data) and feature data are imperative to exploit the ML method, we rationally assigned the class of flavor chemicals and their concentrations as the labeled data. For the feature data of the ML, we allocated 2D scatters coordinating in a space of PCA which is reduced from the 21‐extracted parameters from the transfer curves. Finally, we proceeded with the PCA features to classify the flavor chemicals and predict their concentrations via PCA‐assisted ML, which is a hybridized technique followed by the projection of a discrete regression 3D surface onto a 2D map of classification in the PCA space. Based on the pre‐treatment process by PCA, we could obtain three nontrivial results beyond simply demonstrating the accuracy values of the ML training model. Figure 3b reveals a parallel coordinate plot of the 21 extracted parameters from the transfer curves of all four flavor chemicals encompassing acetic acid, ethyl butyrate, caffeine, and 2‐furanmethanol in Figure 2. Each line in the parallel coordinate plot corresponds to a single point in the multi‐dimensional parameter space, with the lines connecting the values of each parameter to their respective (parallel) axes. This further provides a useful visual indication of the clustering of specific parameters for which the measurement outcomes of the target chemicals are converged, as represented in Figure 3b. For example, it can be readily ascertained that groups of ethyl butyrate possess a converging point at the *V*_th_ of the after‐exposure stage to form a cluster and are distinguished from that of the other flavor chemicals. This signals the capability of valid PCA‐assisted clustering by using the extracted parameters. Based on these salient features, we can conclude that the extracted parameters from the flavor‐chemical dynamics‐related transfer curves are able to facilitate the clustering of the target analytes and visualization of the classification decision boundary through PCA‐assisted ML. This is because these parameters strongly reflect the characteristics of the response behavior of surface‐engineered nanohybrid TFTs for the target analytes. To capture the clustering features of the target chemicals, a PCA was implemented using the 21‐extracted features. PCA is an orthogonal linear transformation that maps data to a new coordinate basis via scalar projection, which serves as a dimensional reduction technique to retain the essence of the data while providing plottable axes through the first two (or three) PCs.^[^ ^42^, ^43^ ^]^ For better visualization, we reduced the multidimensional data‐set of the extracted parameters to a two‐dimensional space (PC1 and PC2). Figure 3c corroborates the resulting PCA plot for the four different types of flavor chemicals. The discernible separation between clusters representing individual flavor chemicals with no overlap enabled the identification of all analytes. This further indicates that PCA is an adequate solution to reduce mass data into two meaningful principal components for a ML dataset. Figure 3d provides the loading scores of each extracted parameter for the two main principal components (PC1 and PC2). A combination of loading scores for the principal components forms a vector revealing their leverage in each principal component, as depicted in Figure 3d. The loading scores explain how variables relate to the two principal components; positive (negative) scores indicate a positive (negative) correlation, highlighting each variable's contribution to explaining these components. The explained variance plot (Figure 3e) of the principal components revealed that the total variance from PC1 (29.2%) and PC2 (22.7%) was approximately 51.9%. Hence, these components contained valuable information from the extracted parameters and are a good reflection of the overall data. To corroborate a scheme to discriminate the four different‐flavor chemicals, we employed a supervised ML approach using the extracted PCs as the ML feature data. Classification can be evaluated with common training algorithms, namely decision tree, linear discriminant, quadratic discriminant, naïve Bayes, support vector machine, k‐nearest neighbor (kNN), ensemble bagged trees, and neural network with the *k*‐fold validation method (*k* = 5). Figure 3f indicates a confusion matrix of 4‐different flavor chemicals from the classification results of the kNN, showing 98.1% prediction accuracy. Figure 3g shows the PCA‐assisted ML results for the decision map of the four different groups by applying the kNN model, which affords clear distinguishable boundaries for acetic acid, caffeine, ethyl butyrate, and 2‐furanmethanol. Consequently, it was able to express the decision boundary map as a function of two‐dimensional PC coordinates with meaningful visibility and the prediction accuracy of the training model. The confusion matrix and classification boundary map with the altered training model are shown in Figures S7 and S8 (Supporting Information), which strongly reflect the characteristics of the classifiers. From these results, it is worth noting that clear visual information can be offered by reducing the dimensionality of the multi‐dimensional parameters of PC characteristics. The accuracies of the different training models are summarized in Figure 3h. The overall training model corroborates superb accuracies showing over 92%. In addition, we compared the performance of the training models by directly utilizing the 21 extracted features (Figure S9, Supporting Information). We found that there is no marked decrease of accuracy due to the limited amount of feature data. This indicates that the PCs strongly dictated and conserved the information of the original 21 extracted parameters (Note S1, Supporting Information). To construct the training model for predicting the analyte concentration, regression models were trained for acetic acid, caffeine, ethyl butyrate, and 2‐furanmethanol using six representative regression models (linear, tree, SVM, ensemble bagging tree, Gauss, and neural network model). To validate the performance of the training model, we carefully divided the data set into training and validation (untrained) categories. The validation dataset was randomly chosen for each concentration. Consequently, we are able to deploy the untrained dataset to verify the validity of the predicted concentration data that has not previously been seen. Supporting Information Figures S10–S13 (Supporting Information) show the predicted concentration data for the four different flavor chemicals from the altered regression training models using a predictive‐concentration regression surface on 2‐dimensional PC spaces (Note S2 and Table S4, Supporting Information). Meanwhile, the Gauss and neural network models showed an extraordinary accuracy of over 99%, we can readily ascertain that the concentration prediction for the untrained data is also included in the corresponding concentration boundary map with 100% accuracy. Based on these results, we can deduce that our PCA‐based pretreatment offered notable visualization of the reduced PC dimension spaces through the classification of target analytes and regression of their concentration, thereby expanding accessible techniques for analyte sensing through ML‐based recognition.

**Figure 4:**
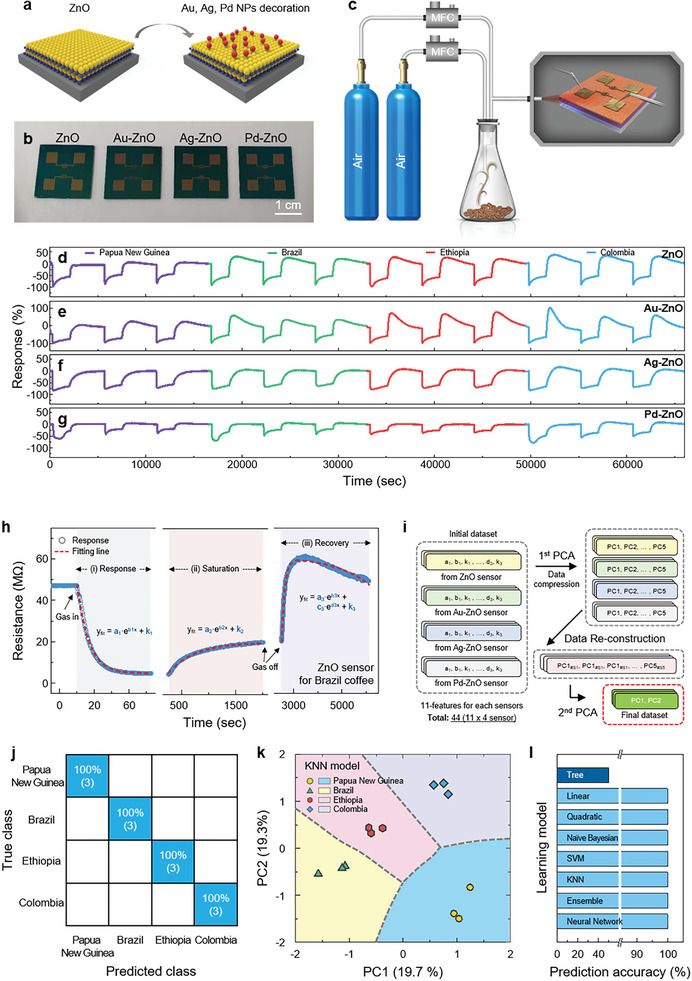
a) Schematic illustration of pristine ZnO thin film and surface‐engineered ZnO thin film by decorating metal NPs. b) Photographs of pristine ZnO and metal NPs‐decorated ZnO. c) Pictorial representation of the measurement setup for gas analytes acquired from coffee beans. Dynamic gas response gained from sensors based on d) pristine ZnO, e) Au NPs‐, f) Ag NPs‐, and g) Pd NPs‐decorated ZnO for four origins of coffee beans (Papua New Guinea, Brazil, Ethiopia, and Colombia‐decaffeine) at 250 °C. (d) Feature extraction from the gas response curve. (Red dashed line; the exponential fitted curve for the response, saturation, and recovery regime). (e) Schematic illustration of the ML data manipulation process. (f) Confusion matrix of the four different coffees from k‐nearest neighbor (kNN) trained model. (g) Decision map for the four different coffees classified by a kNN ML model with k‐fold validation (*k* = 5). h) Summary of the prediction accuracy of the configured training model.

In order to discriminate the aroma of coffee, we deposited ZnO thin films with a thickness of 30 nm by the identical deposition technique used for manufacturing the aforementioned flavor sensor. Subsequently, we employed a thermal evaporator to deposit 3 nm of Au, Ag, and Pd metal nanofilms at a rate of 0.1 A sec^−1^, for the metal‐semiconductor hybridization. These nanohybrids were immediately annealed at 400 °C to agglomerate into metal NPs form, as illustrated in **Figure** **4**a. To attest the gas sensing capabilities, we deposited Cr (3 nm, 0.1 A sec^−1^) / Au (70 nm, 1 A sec^−1^) by a thermal evaporator through a stencil mask without any lithographic fabrication to form interdigitated electrodes. As seen in the photographs (Figure 4b), we can unequivocally ascertain that the device structure was retained without any noticeable structural deformation after the deposition of metal with the annealing process. To explore the morphological evolution of ZnO and metal NPs‐ZnO, we conducted SEM observation, as shown in Figure S14 (Supporting Information), corroborating that the existence of a homogeneous and ultraflat ZnO thin film, while the diverse NPs were distinctly formed after the deposition of three metals. As described above, we can clearly observe that different morphologies are manifested depending on the type of deposited metal. This discrepancy can be accounted for the differences in the temperature‐dependent surface diffusion coefficients and activation energies for agglomeration. Furthermore, the particle size distributions of Au‐ZnO, Ag‐ZnO, and Pd‐ZnO nanohybrids were estimated by SEM observation, as displayed in Figure S14 (Supporting Information). The data were fitted to a log‐normal distribution, resulting in average particle sizes of 9.02, 21.99, and 14.71 nm for Au, Ag, and Pd NPs on ZnO thin films, respectively. To bring coherent morphological information, we further examined the atomic force microscope (AFM) to verify the surface roughness of ZnO, Au‐ZnO, Ag‐ZnO, and Pd‐ZnO, as shown in Figure S15 (Supporting Information). Nearly identical to the SEM observation, the pristine ZnO also possesses homogeneous and continuous surface, whereas the metal NPs were certainly discernible after the deposition and subsequent heat treatment of the nanohybrid. The RMS roughness, a representative parameter for quantifying surface roughness, augments from 1.06 nm for pristine ZnO to 1.681, 4.08, and 4.58 nm after the hybridization with Au, Ag, and Pd metals, respectively. In order to ascertain the chemical signature of ZnO films after the hybridization of manifold metal NPs, we implemented XPS of ZnO and the metal NPs‐ZnO. The representative XPS survey spectra acquired from the ZnO, Au‐ZnO, Ag‐ZnO, and Pd‐ZnO are presented in Figure S16 (Supporting Information). After the deposition of Au, Ag, and Pd on the surface of pristine ZnO, a noticeable decrease in the intensity of the Zn 2*p* and O 1*s* peaks in the constituent core‐level spectra was unequivocally verified. This result apparently links the reduction in the physical area of exposed ZnO on the surface due to the hybridization with metal NPs. Furthermore, we delineated the retention of a chemically identical state associated with the ZnO film irrespective of metal NPs by perceiving marginal peak shifting and persisted spectral lineshape of the Zn 2*p* and O 1*s* bonding states. The presence of pure metal NPs was verified by considering the Au 4*f*, Ag 3*d*, and Pd 3*d* core level spectra. To evaluate the gas response based on the aroma of the coffee beans, we conducted gas reactivity assessments, as presented in Figure 4c. We rationally adopted dry air as the base gas and conducted gas response evaluations for pristine ZnO and nanohybrid‐based gas sensors. The total gas flow rate was configured at 1000 sccm (500 sccm dry air + 500 sccm dry air with bubbled four different coffee beans at 150 °C). Figure 4d–g reveals the dynamic gas response curves of the ZnO‐, Au NPs‐ZnO‐, Ag NPs‐ZnO‐, and Pd NPs‐ZnO‐based sensors at 250 °C for the four different coffee beans (Papua New Guinea, Brazil, Ethiopia, and Colombia‐decaffeine). The gas response was determined by calculating the change in the resistance of the sensors, which is defined as the following equation: gas response (%) = (*R*_gas_−*R*_0_)/*R*_0_ × 100, where *R*_Gas_ denotes the resistance when exposed to the target gas, and *R*_0_ is the resistance in the initial state. The fundamental sensing mechanism of ZnO‐based gas sensors relies on the oxygen adsorption model.^[^ ^44^, ^45^, ^46^, ^47^ ^]^ The rational decoration of noble metals (e.g., Au, Ag, and Pd) promotes electronic and chemical sensitization of sensing materials, which is also called as the spill‐over effect. Consequently, the gas reactivity of the ZnO‐based sensor depends on the types of decorated noble metals owing to the discrepancy in the spill‐over effect, leading to discriminated reactivity of target analytes.^[^ ^48^, ^49^, ^50^, ^51^ ^]^ The estimated gas responses of the ZnO, Au NPs‐ZnO, Ag NPs‐ZnO, and Pd NPs‐ZnO‐based sensors for the four different coffee beans are summarized in Table S5 (Supporting Information). Intriguingly, we further ascertained discernible variations in the gas response which are especially occurred when the coffee vapor was injected or withdrawn. This phenomenon is so‐called ‘overshooting phenomenon’ which is generally occurred due to the various factors, including adsorption or desorption process of the gas molecules on the surface of the sensing materials, diffusion effects, and the presence of moisture or other interferants.^[^ ^52^ ^]^ The overshooting phenomenon in gas sensors refers to a transient response that exceeds the envisaged steady‐state signal when a gas analyte is introduced or removed from the sensing environment. It is characterized by an abrupt increase or decrease in the output signal of sensors, followed by a gradual return to the baseline level. To explore the correlation between the moisture content of the coffee beans and the shape of the gas response curve, we observed the response curve before and after the elimination of moisture by heating the coffee beans at 150 °C with the altering time. As shown in Figure S17 (Supporting Information), we corroborated the photographic images of the heated coffee bean at intervals of 30 min, in which the color of coffee beans became darker accompanied with the loss of their weight from 20.32 to 18.32 g. The gas response of ZnO significantly elevated from −39.14% to −71.58% after the elimination of moisture in the coffee beans owing to the restrained overshooting phenomenon. Nanohybrids based on Au NPs‐ZnO thin film exhibited the largest overshooting effect (*R*_max_‐*R*_sat_ = 125.28%, *R*_sat_‐*R*_min_ = 39.21%), which hints that the Au NPs are the most sensitive to moisture, as demonstrated in Figure S18 (Supporting Information). Based on these results, we ascertained that the four sensors yield distinct electrical responses to the four different coffee beans, facilitated by the ML approach through feature data extraction. In addition, we further characterized and classified the shape and magnitude of each gas response curve into 11 features to incorporate them into ML algorithms to proliferate gas selectivity, as depicted in Figure 4h. To classify the origins of coffee by a surface‐engineered artificial nose (gas sensor), we employed a supervised ML approach by considering the extracted feature data, which were constructed using a PCA‐assisted feature reconstruction approach. The dynamic gas response corroborates the gas injection‐dependent variation in the electrical signals, which indicates that the response curve can further suggest the inherent characteristics of the gas molecule‐sensing surface interplay. To capture the intrinsic features, we specified the dynamic interactions into three‐different stages: i) response, ii) saturation, and iii) recovery region, as depicted in Figure 4h. Then, the individual curves for the response, saturation, and recovery curves can be fitted by the (first‐ or second‐order) exponential growth (or decay) function, as defined below:

(1) $$\mathrm{Fitting}\;\mathrm{curve}=a\;\exp\;\left(b/x\right)+k$$

where *a* is the initial constant, *b* is the time constant, and *k* is the offset. To best fit the detailed features, the recovery curve was fitted by second‐order exponential function, as determined:

(2) $$\begin{array}{ccc} \mathrm{Second}-\mathrm{order}\mathrm{exponential}\mathrm{fitting} & = & a\;\exp\;\left(b/x\right)\\ & & +c\;exp\;\left(d/x\right)+e \end{array}$$

Consequently, we can construct an initial feature dataset consisting of 44 fitting parameters: 11 fitting parameters (response: *a*_1_, *b*_1_, *k*_1_, saturation: *a*_2_, *b*_2_, and *k*_2_ / recovery: a_3_, b_3_, c_3_, *d*_3_, and *k*_3_) each for four‐different sensors (ZnO, Au NPs‐ZnO, Ag NPs‐ZnO, and Pd NPs‐ZnO sensors) for each coffee‐vapor sensing measurements (Figure S19, Supporting Information). Figure 4e illustrates the data manipulation process for ML. First, we carried out a PCA‐driven data‐compression process for the sensing measurements of each sensor; resultantly, eleven‐fitting parameters were compressed into five representative PC scores, as regarded as the new features of the response to the coffee vapors (Figure S20, Supporting Information). To derive 2D coordinates, the initially compressed data were applied second PCA process by organizing the extracted PC scores of all individual sensors into one dataset. Data reconstruction through double PCA possesses two critical merits: i) the response dynamics detected from each sensor are better reflected in the feature parameters for ML through the primary PCA; ii) it is possible to simply express the PC parameters representing each sensor as a new two‐dimensional coordination through the second‐order PCA. The resulting PCA scatter, loading scores, and explained variance plots for the four different origins of coffee measurement are shown in Figure S20 (Supporting Information). To identify the target coffee clusters, we performed supervised ML using the newly derived 2D PCA features. ML classification was assessed with eight representative training models: tree, linear, quadratic, Naïve Bayesian, SVM, kNN, ensemble bagged tree, and neural network model. Figure 4f shows a confusion matrix of the four different target coffees from the classification results of the kNN, showing 100% prediction accuracy. Figure 4g reveals a decision boundary map from the double‐PCA‐assisted ML results, which reveals distinguishable boundaries for the four different coffees from Papua New Guinea, Brazil, Ethiopia, and Colombia‐decaffeine. Figures S21 and S22 (Supporting Information) exhibit the classification boundary map with configured training models and the confusion matrix with modulated classifiers, respectively (Note S3, Supporting Information). The prediction accuracies of the training models are summarized in Figure 4h. Except for the tree classifier, all the training models exhibited 100% prediction accuracy. From these results, we demonstrate that the extracted PCs strongly reflect the interfacial dynamics between the gas molecules and functionalized sensing surfaces. Thus, the PCA‐assisted data organization process renders excellent distinguishable features, which are mostly undetectable due to slight electrical changes. In addition, to compare the prediction accuracy, we compared the ML performance with single‐PCA‐configured features (Figure S23, Supporting Information). Figures S24 and S25 (Supporting Information) display the decision boundary map and the confusion matrix plot for the configured classification models with a single‐PCA feature dataset. The prediction accuracy of ML is summarized in Figure S26 (Supporting Information), which suggests that the double‐PCA approach provides more discernible characteristics for classifiers than the single‐PCA features. These salient features are attributed to the five initially extracted PC scores that accurately reflect the sensing behavior of each ZnO‐based sensor (Figure S27, Supporting Information).

## 3 Conclusion

We developed an innovative sensor system, the artificial Q‐grader comprising surface‐engineered metal nanoparticles/ZnO and organic polymer/ZnO thin films, as well as AI‐based statistical data analysis, for dual discrimination of gas and liquid analytes. This system successfully mimics the human olfactory and gustatory systems, enabling multifaceted IoT sensor applications. The ML model for gas and liquid classification and PCA‐assisted regression exhibited high prediction accuracy and readability. The AI model employed in this study precisely analyzed and visually represented the data, extracting 21 features from the liquid sensor and a comprehensive set of 44 parameters from the four distinct gas sensors. Our findings highlight the substantial potential for advancing future data analysis technologies by replacing artificial human sensing platforms. Furthermore, the efficacy of the sensor system was demonstrated by discerning the aroma and flavor of coffee beans originating from Papua New Guinea, Brazil, Ethiopia, and Colombia‐decaffeine. We suggest that the artificial Q‐grader represents a significant advancement in personalized and portable sensors, thus paving the way for applications in environmental monitoring, health diagnosis, and food quality assessment. The integration of hybrid nanomaterials and ML offers unparalleled possibilities for future sensor technologies.

## 4 Experimental Section

### Fabrication of PVDF‐HFP/ZnO Hybrid Film‐Based TFTs Sensors

A heavily doped p‐type Si substrate with 300 nm‐thick thermally grown SiO_2_ was used as a bottom gate electrode and gate dielectric layer. For ZnO TFTs, a 15 nm‐thick ZnO active layer was deposited on the SiO_2_/Si substrates by atomic layer deposition (ALD). The ALD process progressed with diethyl zinc (DEZ) and H_2_O pulses for 10 s and N_2_ purges for 1 s, repeating two times. This process was repeated for 85 cycles at 100 °C. The Al interdigitated electrodes as source‐drain electrodes, were deposited by thermal evaporation to a thickness of 60‐nm through a shadow mask, which has a channel width (W) of 1000 µm and length (L) of 100 µm. For PVDF‐HFP/ZnO hybrid film, PVDF‐HFP pellets (0.05 wt.%, Sigma–Aldrich, Mw ≤ 455 000; Mn ≤ 110 000) were stirred at room temperature for ≈2 days to dissolve in N, N‐Dimethylformamide (Sigma–Aldrich, 99.8% purity). Then, the PVDF‐HFP solution was spin‐coated on top of the ZnO active layer at 6000 rpm for 60 s. After this processing, the sample was dried in air at 80 °C for 1 h to enhance the interfacial adhesion and remove the residual solvent. To measure electrical characteristics, a polydimethylsiloxane (PDMS) block was used to expose the liquid‐phase analytes only to the electrode channel of polymer‐functionalized ZnO TFTs.

### Fabrication of Pristine ZnO, Au NPs‐ZnO, Ag NPs‐ZnO, and Pd NPs‐ZnO‐Based Sensors

Gas sensors based on ZnO and metal NPs/ZnO nanohybrids were fabricated via three‐step processes. First, 30 nm‐thick ZnO thin films were deposited using ALD onto SiO_2_ (300 nm)/Si(001) substrates. The ALD process was performed using the optimal conditions to fabricate the aforementioned TFT sensors, with the exception of the number of ALD cycles, which was increased to 159 cycles. Second, three nm‐thick Au was deposited, Ag and Pd nanofilms by thermal evaporation with the deposition rate of 0.1 Å sec^−1^. Finally, the nanohybrid films were immediately annealed at 400 °C under Ar atmosphere to agglomerate in a metal NP form. To evaluate the sensing performance, thermal evaporation was employed to deposit interdigitated electrodes with the thickness of Cr (3 nm, 0.1 Å sec^−1^)/Au (70 nm, 1 Å sec^−1^) through a shadow mask.

### Preparation of Analytes Associated with Coffee Beans

In this study, coffee taste chemicals were determined by the amount of caffeine (Bitterness), acetic acid (Sourness), and 2‐furanmethanol and ethyl butyrate (Sweetness), based on gas chromatography‐mass spectrometry (GC‐MS) data of four roasted coffee beans (Papua New Guinea, Brazil, Ethiopia, and Colombia‐decaffeine). All of taste analytes (Sigma–Aldrich) were dispersed at a concentration of 1 m in DI water using ultra‐sonication for 1 h at room temperature. Then, the mixture solution was diluted in DI water up to a low concentration to prepare the taste analyte solutions. To assess the gas response based on the aroma of coffee beans, dry air (80% of N_2_ + 20% of O_2_, Chung‐ang Industrial Gas) was utilized as both the base gas and bubbling agents. To investigate the gas reactivity in the setup similar to the GC‐MS experimental conditions, coffee beans were heated to 150 °C using a hot plate and conducted bubbling by introducing air through the Erlenmeyer flask, as displayed in Figure S28 (Supporting Information). The total gas flow rate was configured at 1000 sccm (500 sccm dry air + 500 sccm coffee aroma).

### Characterization

The chemical identification of ZnO films with and without PVDF‐HFP were investigated by attenuated total reflectance spectrometer (ATR, Alpha‐P & Alpha‐T, Bruker), and X‐ray photoelectron spectroscopy (XPS, K‐Alpha, Thermo Fisher Scientific). The surface morphologies and thicknesses of ZnO thin films and PVDF‐HFP polymer‐functionalized ZnO layers were characterized by atomic force microscopy (AFM, Innova, Bruker) and a field emission scanning electron microscope (FE‐SEM, JSM‐6700F, Jeol). The gas sensing measurements were conducted by a customized gas characterization system (≈1111 cm^3^, MSTECH) integrated with a gas flow controller (SR312, Bronkhorst High‐tech) and an electric source meter (Keithley 2612B, Keithley). All the gas sensing measurements were performed at 250 °C under ambient pressure with an applied bias of 1 V.

### Machine Learning

The prepared dataset was employed to train the ML model with a primary focus on predicting both 4‐different types of taste chemicals and 4‐different types of coffee vapors. Using MATLAB, the dataset was integrated with various classification and regression models, including linear, tree, SVM, ensemble bagged tree, Gauss, and neural network models. To ensure robustness and mitigate overfitting, a *k*‐fold (*k* = 5) validation approach was implemented during training. Using this MATLAB‐based approach, the performances of these models were rigorously evaluated by employing metrics such as the RMSE, MAE, and R^2^ values to assess their accuracies and predictive capabilities.

## Conflict of Interest

The authors declare no conflict of interest.

## Supporting information

Supporting Information

## Data Availability

Research data are not shared.
