# Supplementary material for: Artificial Q‐Grader: Machine Learning‐Enabled Intelligent Olfactory and Gustatory Sensing System
Source: Adv Sci (Weinh). 2024 Apr 6;11(23):2308976. doi: 10.1002/advs.202308976 (PMC11186046; doi:10.1002/advs.202308976)
Supplement: Supplementary file 1 — Supporting Information [file ADVS-11-2308976-s001.pdf]

ADVANCED SCIENCE
Open Access

Supporting Information

for *Adv. Sci.*, DOI 10.1002/advs.202308976

Artificial Q-Grader: Machine Learning-Enabled Intelligent Olfactory and Gustatory Sensing System

Moonjeong Jang, Garam Bae, Yeong Min Kwon, Jae Hee Cho, Do Hyung Lee, Saewon Kang, Soonmin Yim, Sung Myung, Jongsun Lim, Sun Sook Lee, Wooseok Song and Ki-Seok An**

Supporting Information

Artificial Q-grader: Machine learning-enabled intelligent olfactory and gustatory sensing system

Moonjeong Jang, Garam Bae, Yeong Min Kwon, Jae Hee Cho, Do Hyung Lee, Saewon Kang, Soonmin Yim, Sung Myung, Jongsun Lim, Sun Sook Lee, Wooseok Song and Ki-Seok An**

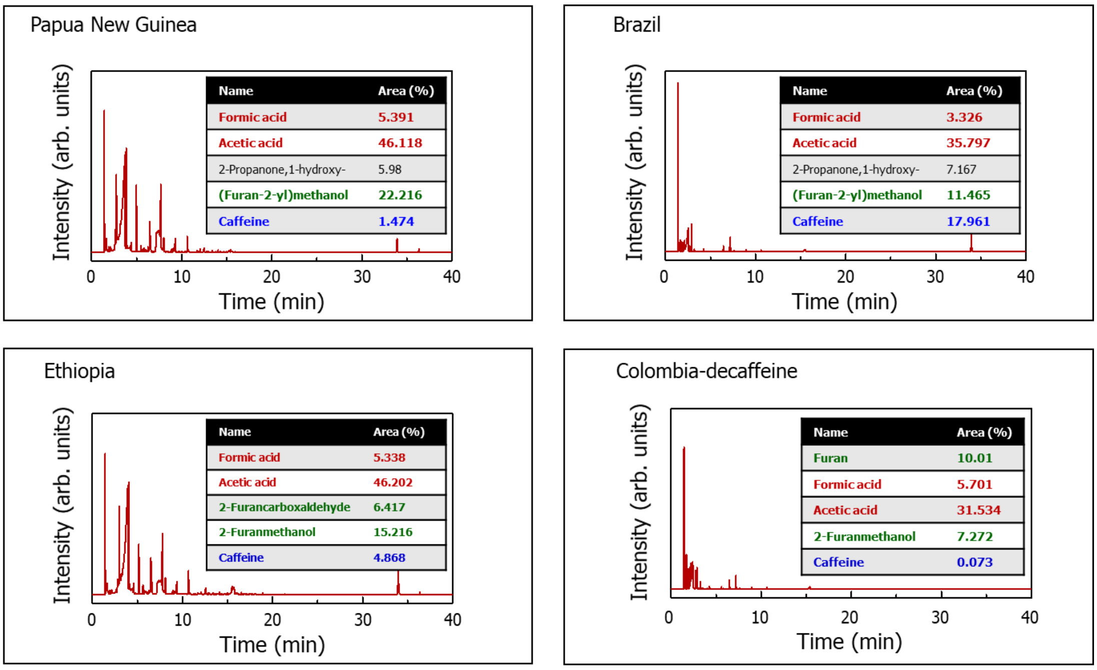

Figure S1. GC-MS spectra coupled with proportion of flavor chemicals extracted from four different coffee beans (Papua New Guinea, Brazil, Ethiopic, and Colombia-decaffeine).

Instruments	Agilent Technologies 7890A gas chromatograph / LECO PEGASUS BT Time of Flight mass spectrometer (GC-TOF-MS)	
**Column**	DB-5MS (30 m × 0.25 mm × 0.25 μm) Capillary	
**Carrier gas**	Gas type	He gas
	Flow rate	1 ml/min
**Injector**	Temperature	200 ºC
	Injection mode	Split
	Split ratio	80 : 1
**Oven**	Oven temperature	40 ºC (3 min) to 240 ºC (3 min)
	Rate	5 ºC/min
**Ionization mode**	Electron voltage	70 eV
	Detector	Electron multiplier / 2129 V
	Analytical mode	Time of Flight
	Scanning range	40 ~ 600 m/z
**Headspace**	Mass	Coffee bean 0.5 g
	Temperature	150 ºC
	Time	30 min

Table S1. Summarized measurement conditions for GC-MS of four different coffee beans (Papua New Guinea, Brazil, Ethiopia, and Colombia-decaffeine).

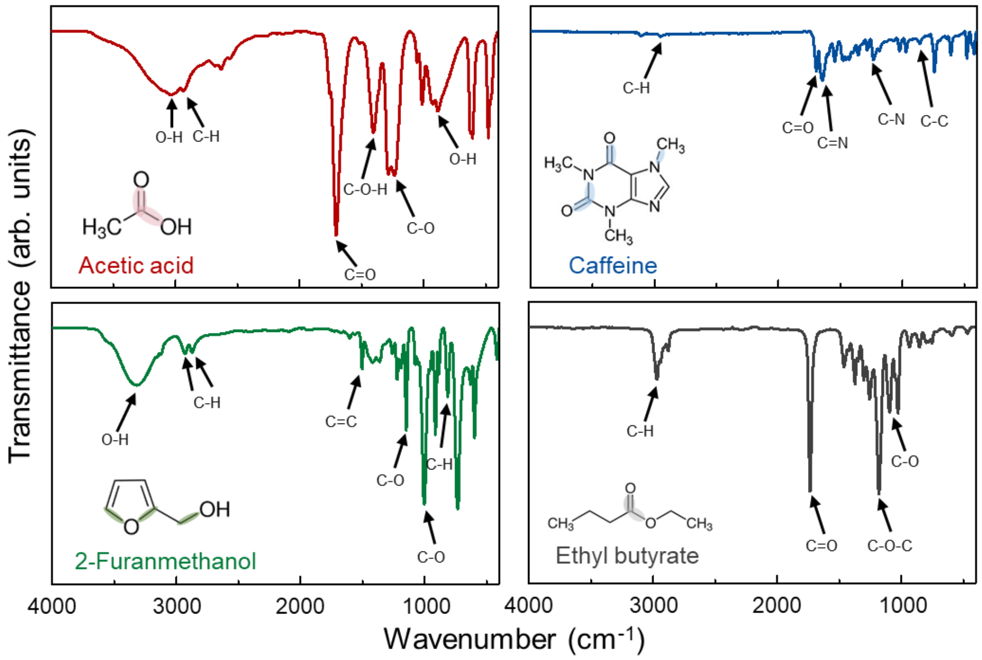

Figure S2. FTIR-ATR spectra for flavor compounds in dilute aqueous solutions of acetic acid (sourness), caffeine (bitterness), 2-furanmethanol and ethylbutyrate (sweetness).

Coffee chemicals	Wavenumber (cm^{-1})	Band assignment
**Acetic acid**	2500 - 3300	O-H stretching
	2942	C-H stretching
	1703	C=O stretching
	1410	C-O-H in-plane bending
	1234	C-O stretching
**Caffeine**	2954, 3111	C-H stretching
	1695	C=O stretching
	1646	C=N stretching
	1230	C-N stretching
	855	C-C stretching
**2-Furanmethanol**	3200 - 3500	O-H stretching
	2872, 2934	C-H stretching
	1502	C=C stretching
	1004, 1148	C-O stretching
	814	C-H stretching
**Ethyl butyrate**	2967	C-H stretching
	1732	C=O stretching
	1181	C-O-C bending
	1094	C-O stretching

Table S2. Summarized band assignments of FTIR-ATR spectra for chemicals (acetic acid, caffeine, 2-furanmethanol, ethylbutyrate).

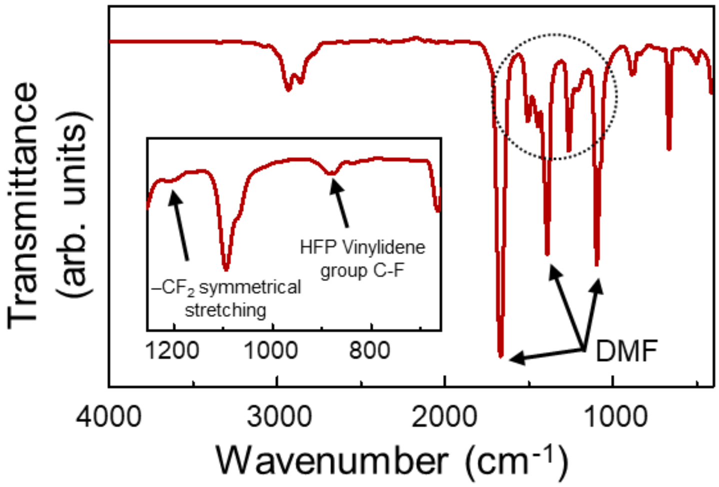

Figure S3. FTIR-ATR spectrum of PVDF-HFP solution prepared in DMF.

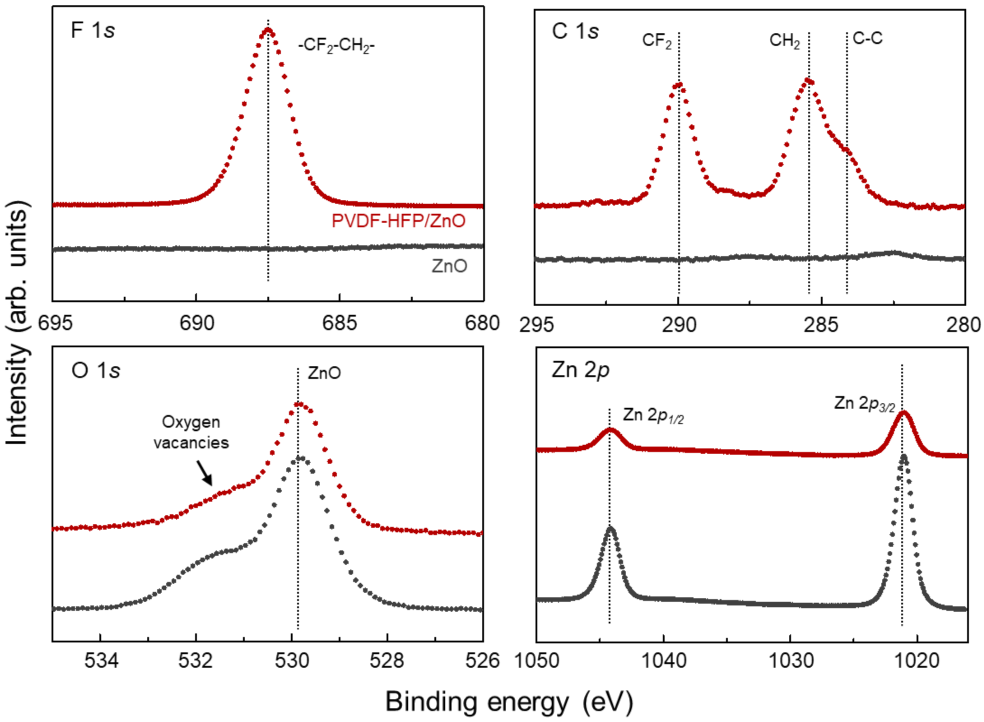

Figure S4. F 1*s*, C 1*s*, O 1*s* and Zn 2*p* core level XPS spectra of ZnO thin films and PVDF-HFP/ZnO thin films.

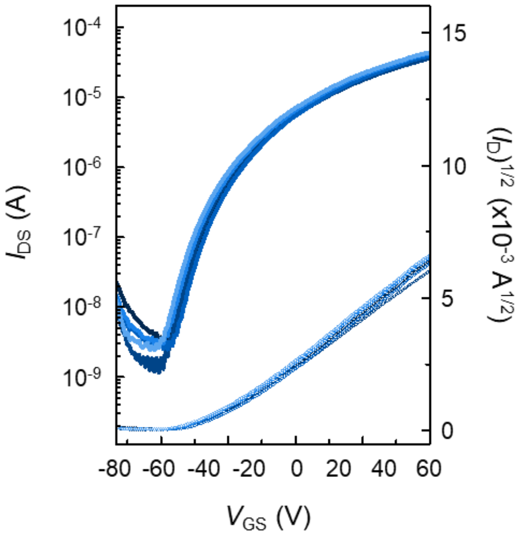

Figure S5. Transfer characteristics of surface-engineered TFT-based sensors at initial state (before exposure).

I_{ON} (A)	I_{OFF} (A)	I_{ON}/I_{OFF}	Carrier mobility (cm^2/Vs)	SSwing (V/dec)	V_{Th} (V)
4.1 x 10^{-5}	3.3 x 10^{-9}	1.3 x 10^{4}	0.226	6.54	-37.20
3.6 x 10^{-5}	1.2 x 10^{-9}	3.1 x 10^{4}	0.201	5.18	-40.54
4.0 x 10^{-5}	2.5 x 10^{-9}	1.6 x 10^{4}	0.220	5.71	-35.48
4.3 x 10^{-5}	2.8 x 10^{-9}	1.5 x 10^{4}	0.238	6.16	-38.46
4.4 x 10^{-5}	2.4 x 10^{-9}	1.8 x 10^{4}	0.247	6.43	-39.80

Table S3. Extracted performance parameters from the transfer characteristics in Fig. S5.

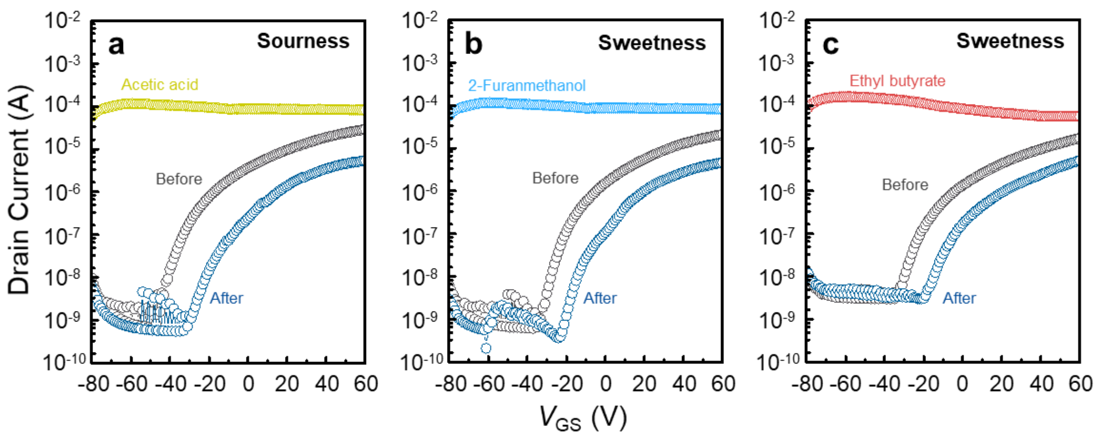

Figure S6. Transfer characteristics of surface-engineered TFT-based sensors before and after exposure, and exposing to the (a) acetic acid, (b) 2-furanmethanol and (c) ethyl butyrate solution.

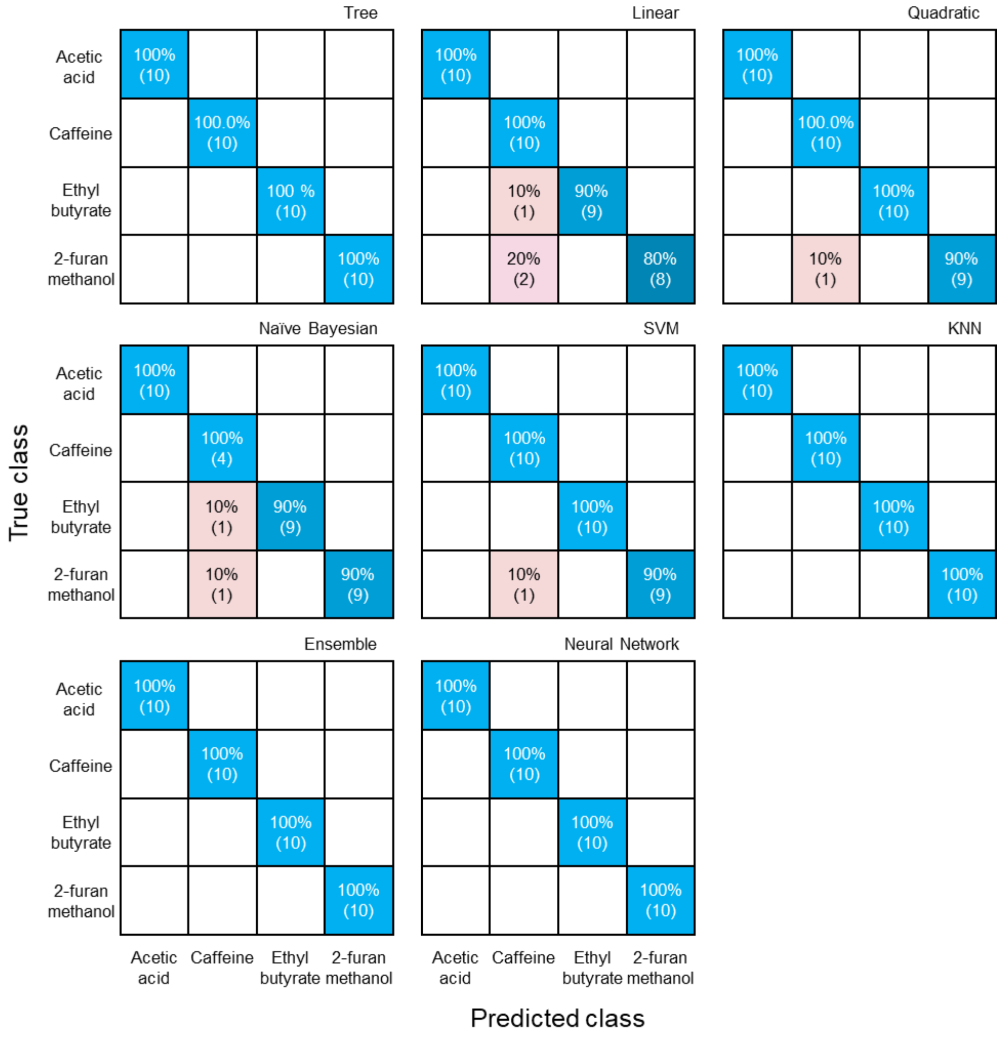

Figure S7. Confusion matrix of modulated classifiers such as decision tree model, linear discriminant model, quadratic discriminant model, Naïve Bayes model, support vector machine, k-nearest neighbor, ensemble bagged trees, and neural networks.

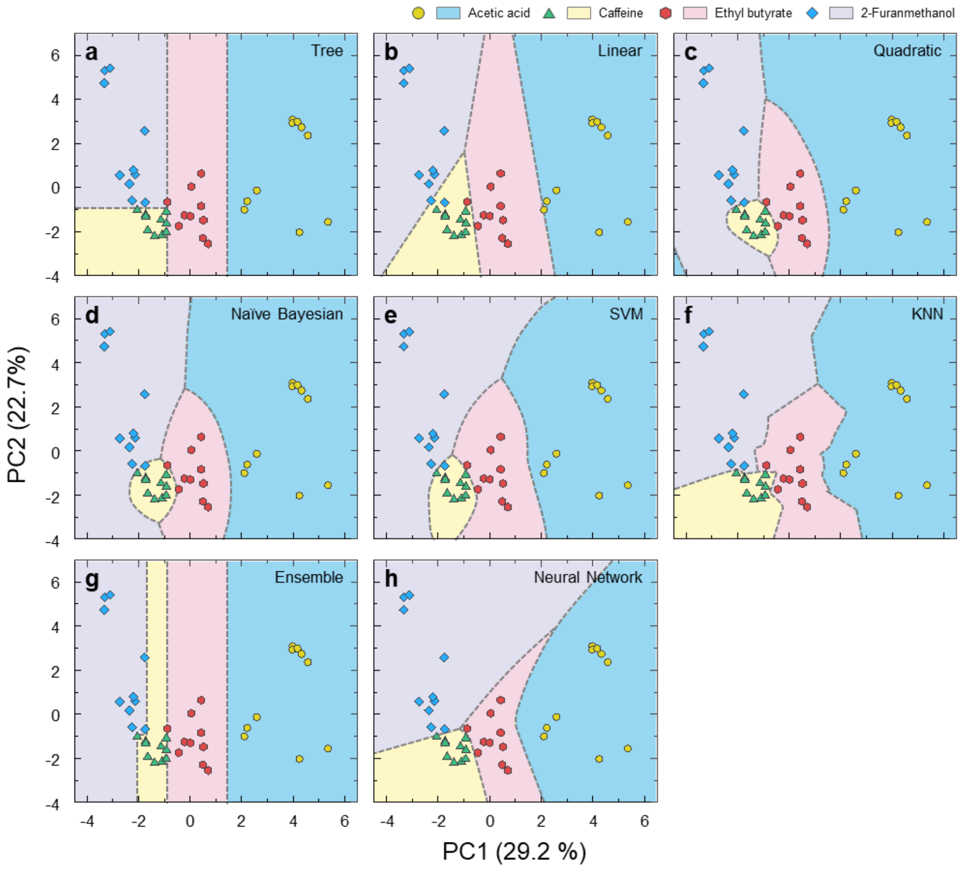

Figure S8. Classification boundary map with configured training model with strongly reflecting the characteristics of the classifier for (a) decision tree model, (b) linear discriminant model, (c) quadratic discriminant model, (d) Naïve Bayes model, (e) support vector machine, (f) k-nearest neighbor, (g) ensemble bagged trees, and (h) neural networks.

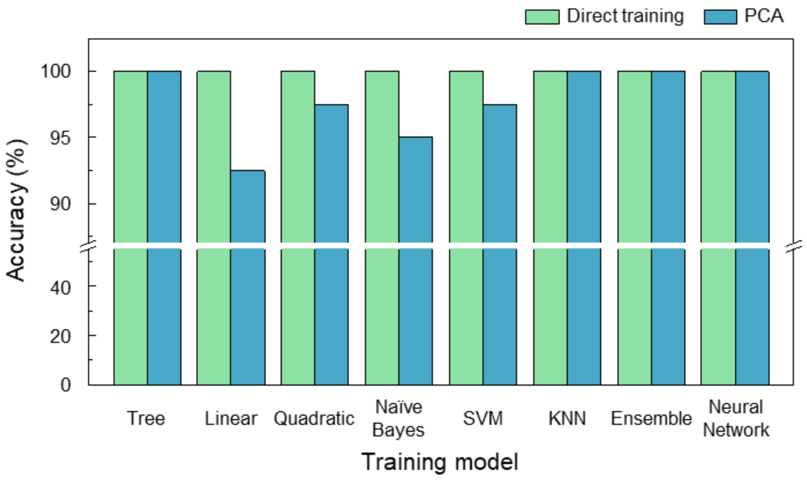

Figure S9. Summarized accuracy of altered training model by utilizing the original 21-extracted features and PCA features.

Supplementary Note 1.

Visualization of decision boundary map for classification on PC spaces.

Recently, in terms of explainable (or interpretable) ML, it is indispensable to propose a new methodology that facilitates an intuitive interpretation for intuitive understanding why this decision was made, rather than simply presenting their scores. Along these lines, a visualization of ML results gives a great opportunity that enable to compare, analysis, and observe unique characteristics between the various types of training models. Principal component analysis (PCA) has been widely adopted for not only a common classifier as an unsupervised ML but also data compression by reduction of dimensionality. In this study, by utilizing principal component analysis (PCA), a data pretreatment, which reduces the classes expressed by 21-TFT-characteristic parameters to two PC coordinates, is performed as an intermediate step between the data acquisition process and ML (classification and regression). Through applying this pre-treatment process by PCA, we could obtain three nontrivial results beyond simply demonstrating the accuracy values of the ML training model; i) it is able to express the decision boundary map as a function of two-dimensional PC coordinates (Figure 3g and Supporting Information Figure S8, S10-13). ii) We can compare the inherent and unique characteristics of various training models in visualized decision boundary map (Supporting Information Figure S8, S10-13).

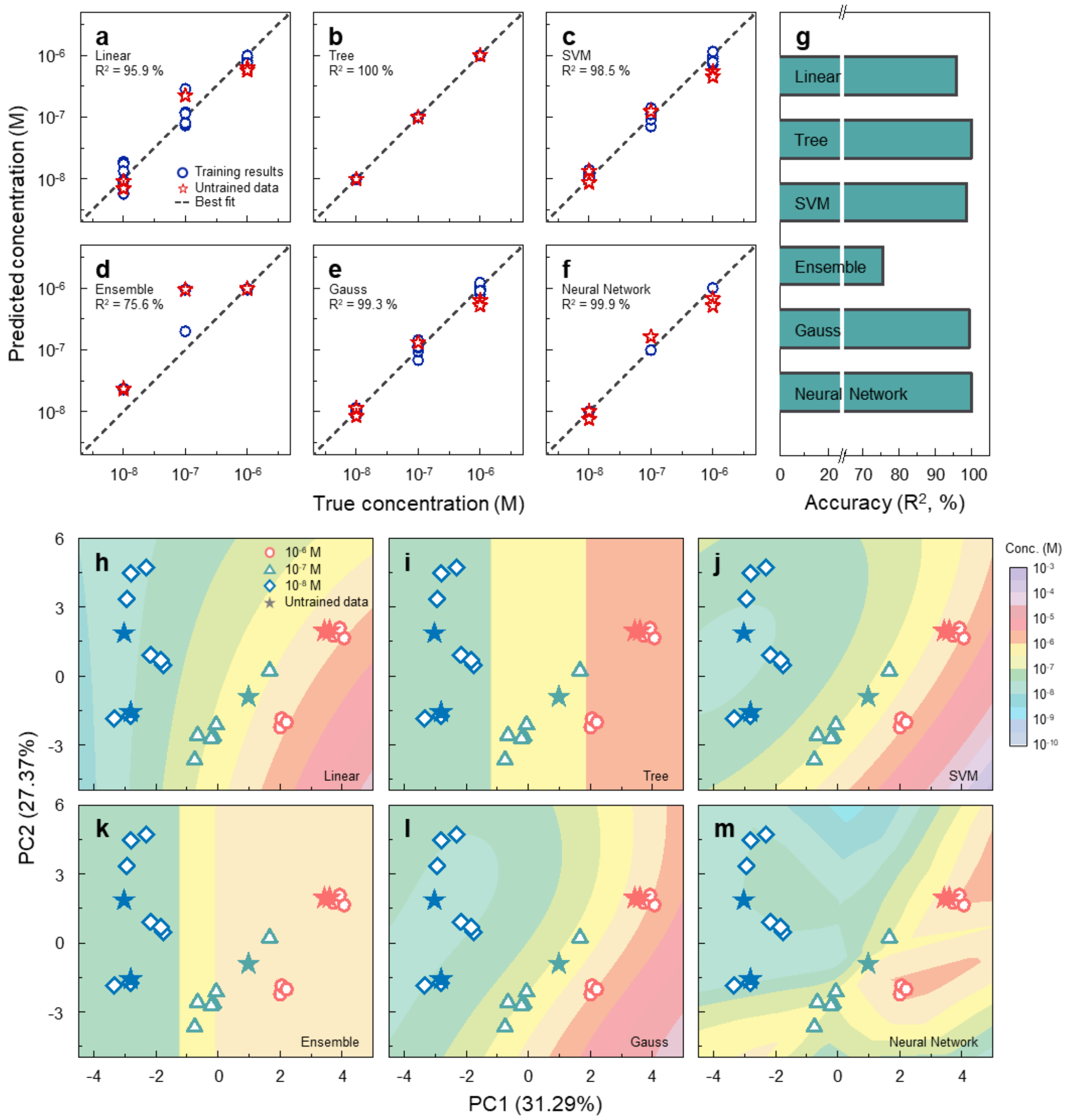

Figure S10. Regression results for acetic acid. (a-f) Relationships of predicted analyte concentration vs. real analyte concentration for 10^{-6} – 10^{-7} M of acetic acid by applying linear regression model, tree model, SVM model, ensemble bagged tree model, Gauss model, and neural network model. (g) Summary of accuracy for the 6-different training model. (h-m) Predicted-concentration regression surface in 2-dimensional PCs space for the altered model.

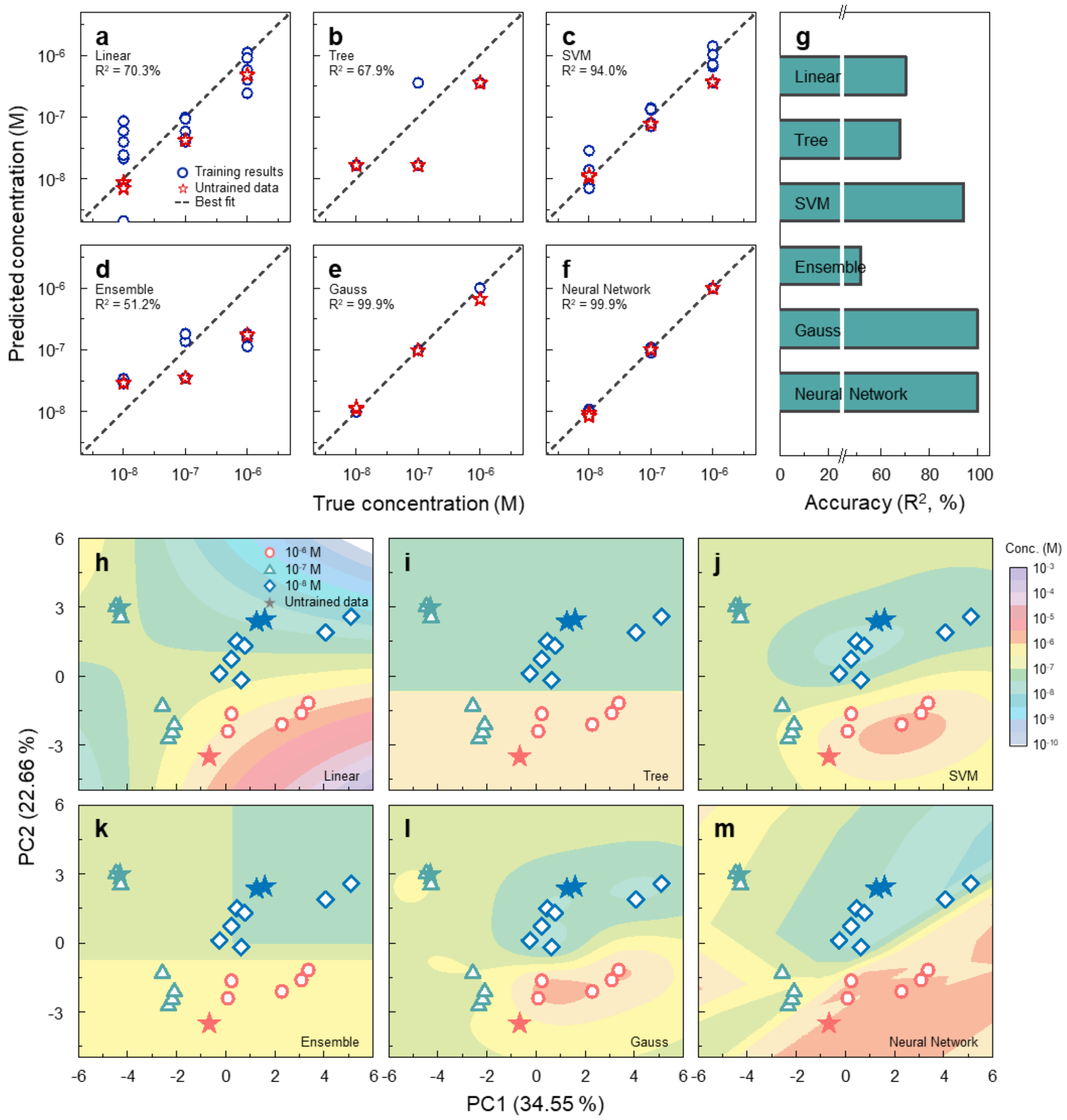

Figure S11. Regression results for ethyl butyrate. (a-f) Relationships of predicted analyte concentration vs. real analyte concentration for $10^{-6} - 10^{-7}$ M of acetic acid by applying linear regression model, tree model, SVM model, ensemble bagged tree model, Gauss model, and neural network model. (g) Summary of accuracy for the 6-different training model. (h-m) Predicted-concentration regression surface in 2-dimensional PCs space for the altered model.

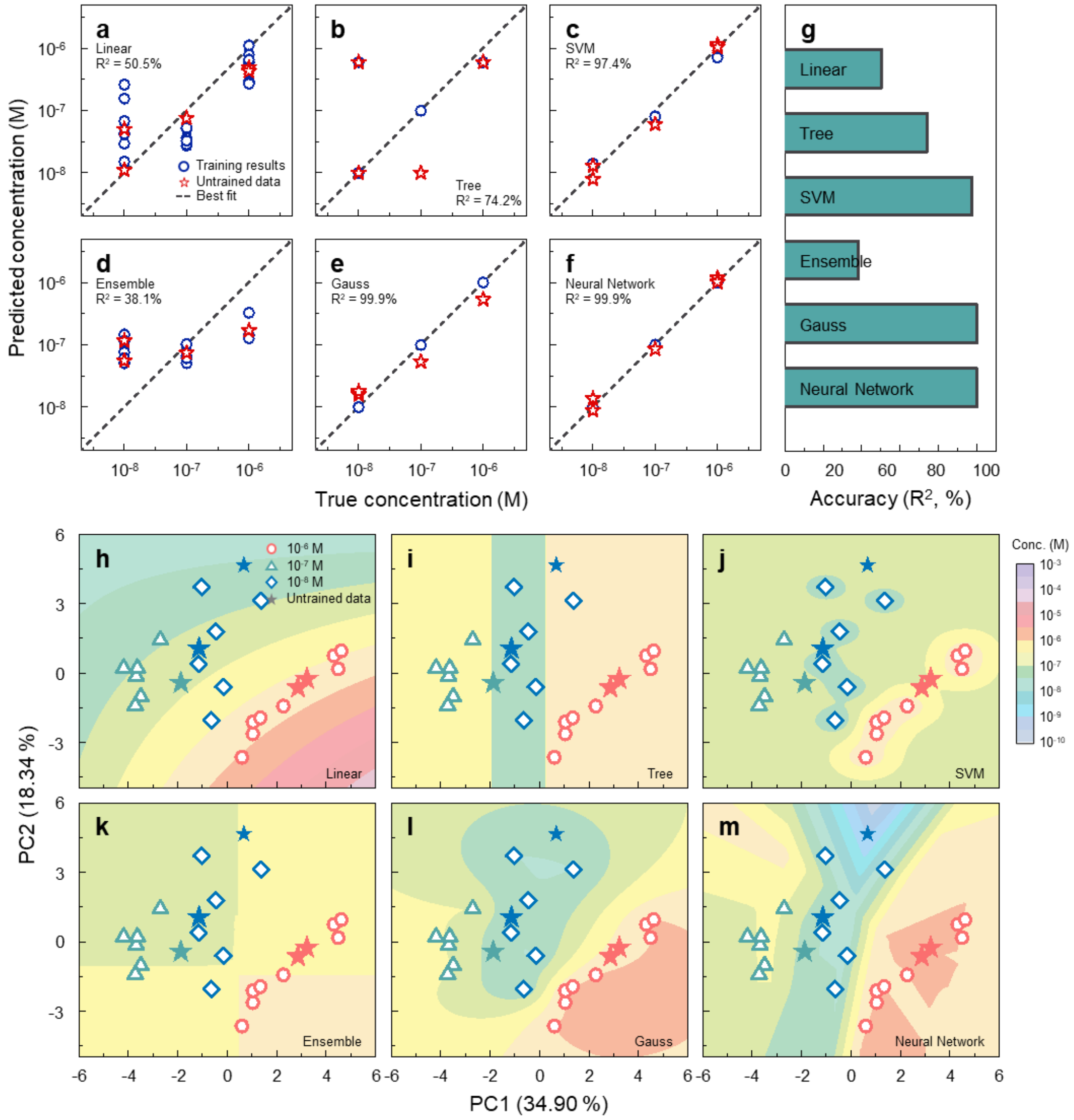

Figure S12. Regression results for caffeine. (a-f) Relationships of predicted analyte concentration vs. real analyte concentration for 10^{-6}–10^{-7} M of acetic acid by applying linear regression model, tree model, SVM model, ensemble bagged tree model, Gauss model, and neural network model. (g) Summary of accuracy for the 6-different training model. (h-m) Predicted-concentration regression surface in 2-dimensional PCs space for the altered model.

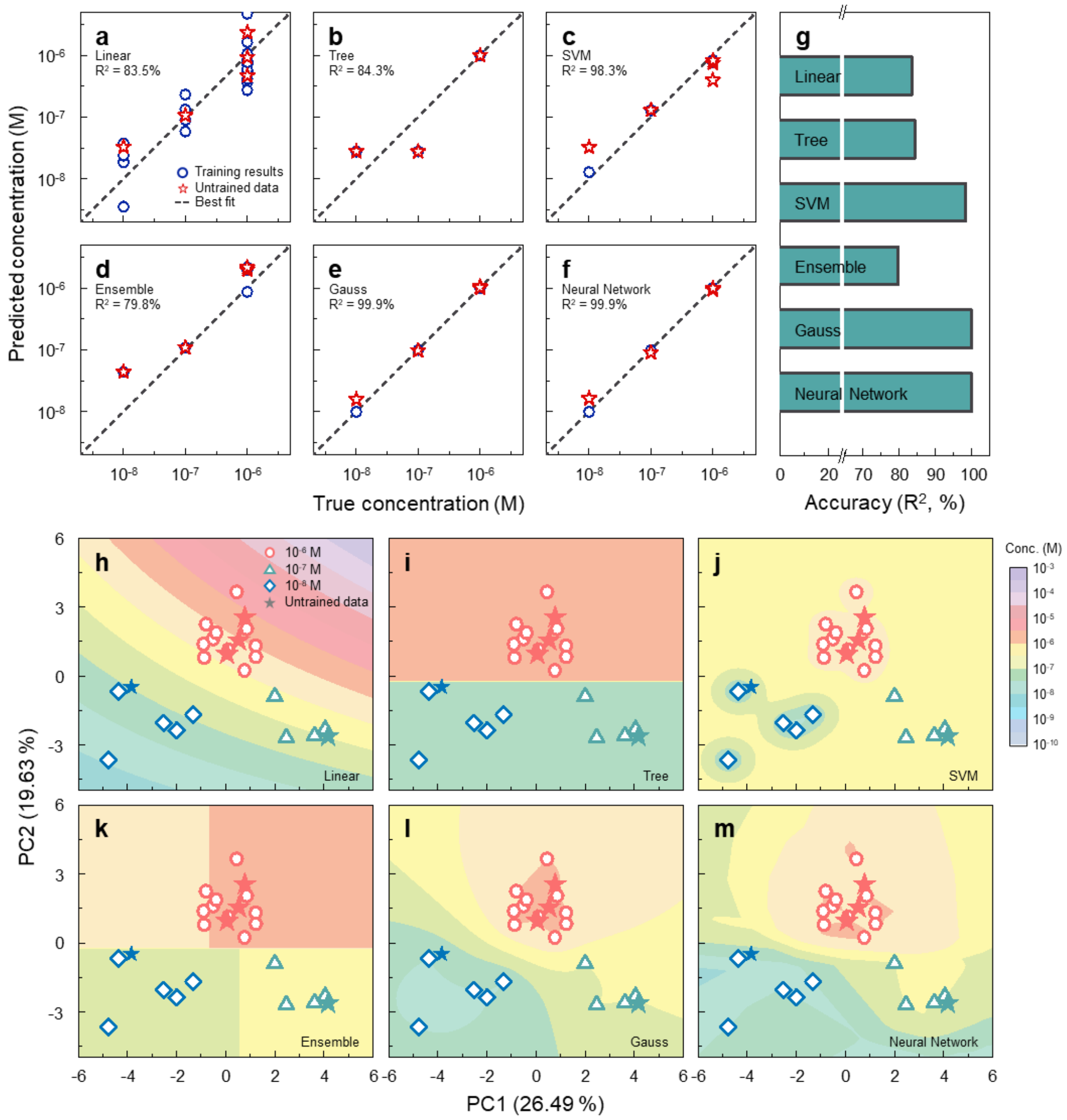

Figure S13. Regression results for 2-furanmethanol. (a-f) Relationships of predicted analyte concentration vs. real analyte concentration for 10^{-6} – 10^{-7} M of acetic acid by applying linear regression model, tree model, SVM model, ensemble bagged tree model, Gauss model, and neural network model. (g) Summary of accuracy for the 6-different training model. (h-m) Predicted-concentration regression surface in 2-dimensional PCs space for the altered model.

Supplementary Note 2.

Training regression model for flavor chemicals

To establish the training model for predicting the analyte concentration, regression models are trained for acetic acid (Supporting Information Figure S10), ethyl butyrate (Supporting Information Figure S11), caffeine (Supporting Information Figure S12), and 2-furanmethanol (Supporting Information Figure S13) using 6-representative regression model (linear, tree, SVM, ensemble bagging tree, Gauss, and neural network model) with k-fold (k = 5) validation method. To assess the performance of the regression model for newly importing data, we carefully divide the data set into two categories for training and validation (untrained). The validation data set is randomly chosen for each concentration. The results of regression model are represented in Supporting Information Figure S10-13, including plots of predicted concentration *vs.* real concentration for each regression models (a-f), a summary of prediction accuracy for training models (g), regression surfaces of predicted-concentration (h-m). From the predicted concentration plots, trained data (blue circle) is likely to be fitted and clustered along the best-fitting line, which indicates our regression models are well-trained with the newly-organized PC scores under the PCA. Furthermore, we suggest that our training models are able to predict the concentration for untrained data (red star) correctly. The accuracy (R^2) can be calculated using the residual scatter from the fitting line (black dashed line) as follows:

$$Accuracy(R^2, \%) = 1 - \frac{\sum(y-p)^2}{\sum(y-\bar{y})^2}$$

where y is the real value, p is the predicted value, and $\bar{y}$ is the mean of the real value at the specified corresponding analyte concentration. Then, $y-p$ can be described as a residual scatter from the prediction of the real value through the machine learning results. $y-\bar{y}$ is the total variance. In general, because a higher R^2 leads to a well-fitted model compared with the

real values, the accuracy of the training model can be estimated using the R^2 value. The ML results exhibit a high-scored accuracy with Gauss and neural network regression model. The scored accuracies are summarized in Supporting Information Table S8, as followed;

**Chemicals**	**Linear**	**Tree**	**SVM**	**Ensemble**	**Gauss**	**Neural network**
Acetic acid	95.9 %	100 %	98.5 %	75.6 %	99.3 %	99.9 %
Ethyl butyrate	70.3 %	67.9 %	94.0 %	51.2 %	99.9 %	99.9 %
Caffeine	50.5 %	74.2 %	97.4 %	38.1 %	99.9 %	99.9 %
2-furan methanol	83.5 %	84.3 %	98.3 %	79.8 %	99.9 %	99.9 %

Table S4. Prediction accuracy of training models for the concentration of acetic acid (Fig. S10), ethyl butyrate (Fig. S11), caffeine (Fig. S12) and 2-furanmethanol (Fig. S13).

Similar to the represented decision boundary map for classification models, the regression surface of predicted concentration can be displayed in 2-dimensional principal-components (PC) spaces for each training model (Supporting Information Figure S10-13). From the regression surface map, we can readily ascertain that the concentration prediction for untrained data is also included in the corresponding concentration boundary perfectly.

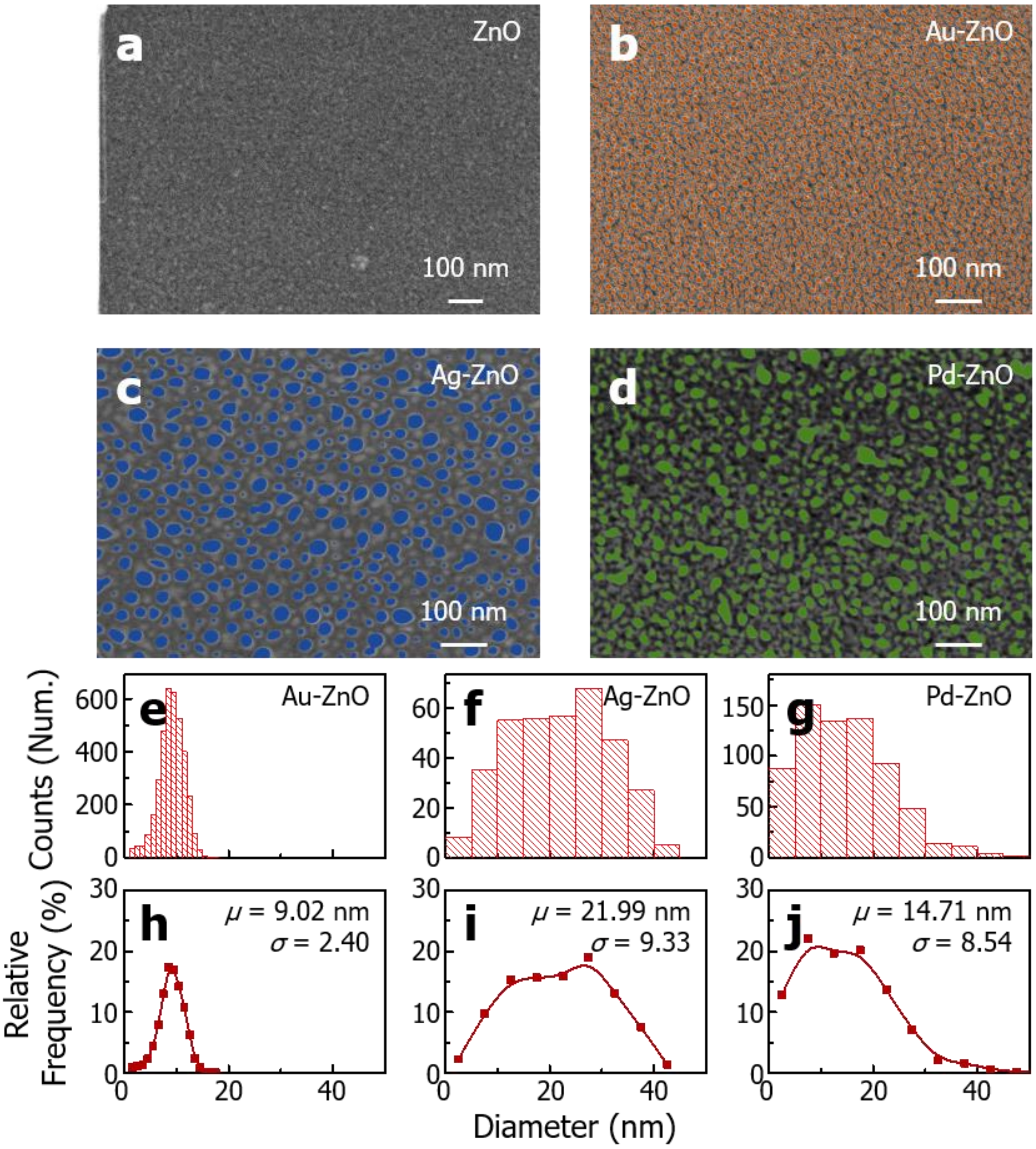

Figure S14. Representative SEM top-view images of (a) pristine ZnO, (b) Au-ZnO, (c) Ag-ZnO, and (d) Pd-ZnO. Particle size distribution histogram and Gaussian fitting extracted from SEM images of (e, h) Au-ZnO, (f, i) Ag-ZnO, (g, j) Pd-ZnO.

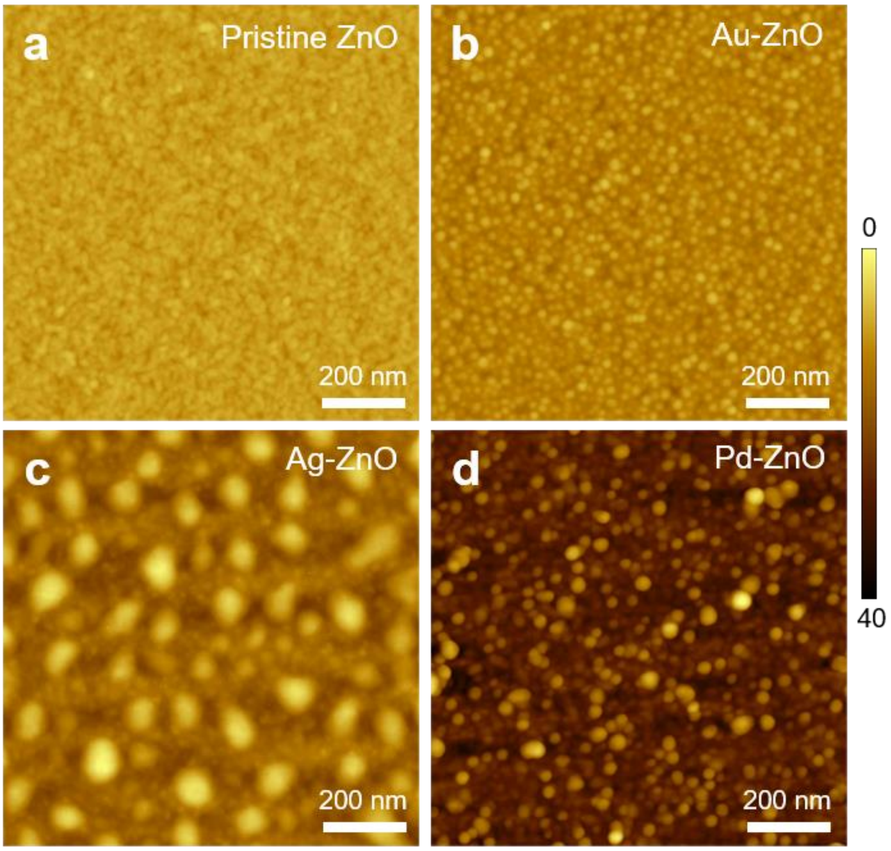

Figure S15. Representative AFM images acquired from (a) pristine ZnO, (b) Au-ZnO, (c) Ag-ZnO, and (d) Pd-ZnO.

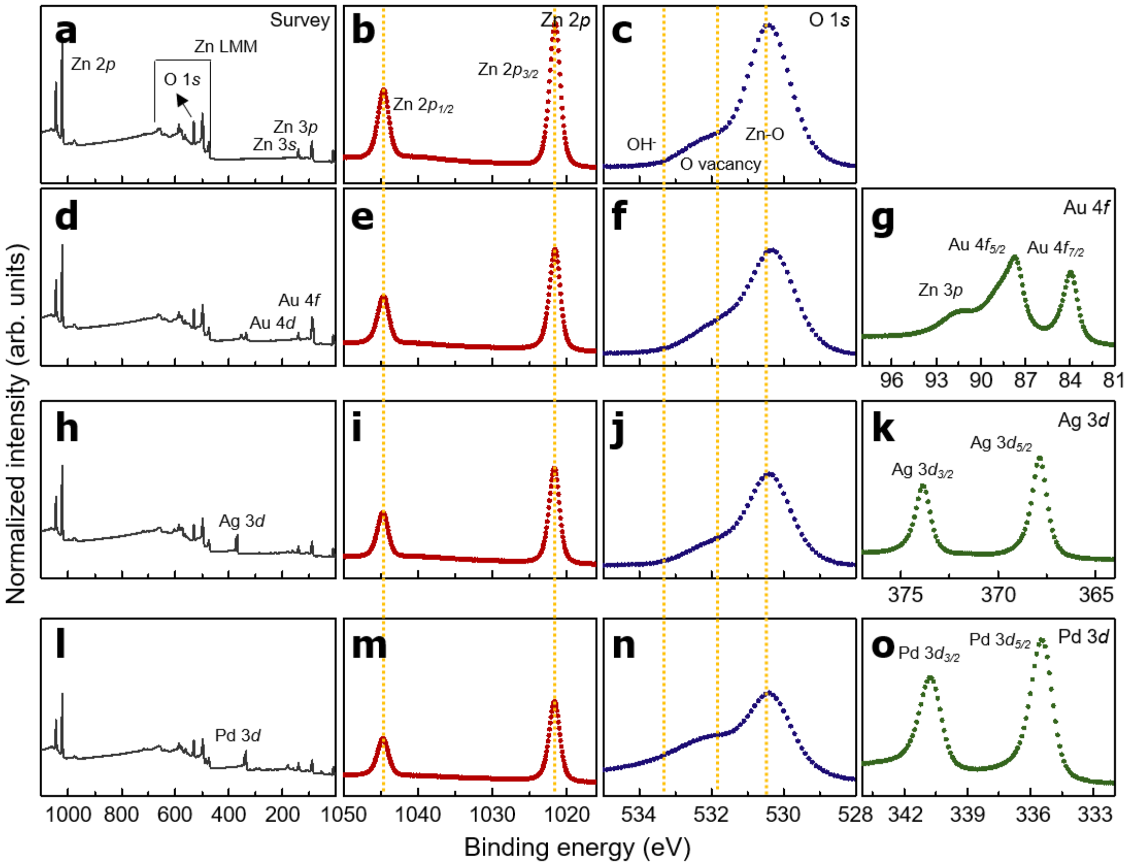

Figure S16. Chemical identification of pristine ZnO and metal-funtionalized ZnO by XPS. Survey, Zn 2*p*, O 1*s*, Au 4*f*, Ag 3*d*, Pd 3*d* core level spectra acquired from (a-c) pristine ZnO, (d-g) Au-coated ZnO, (h-k) Ag-coated ZnO, and (l-o) Pd-coated ZnO, respectively.

	ZnO	Au-ZnO	Ag-ZnO	Pd-ZnO
Papua New Guinea	-45.76 %	-51.92 %	-56.05%	-52.59 %
Brazil	-68.92 %	-51.70 %	-56.59 %	-62.03 %
Ethiopia	-59.26 %	-56.10 %	-59.47 %	-59.66 %
Colombia	-19.44 %	-21.74 %	-24.95 %	-34.99 %

Table S5. Summarized gas responses acquired from ZnO, Au NPs-ZnO, Ag NPs-ZnO, and Pd NPs-ZnO based gas sensors for four types of coffee beans.

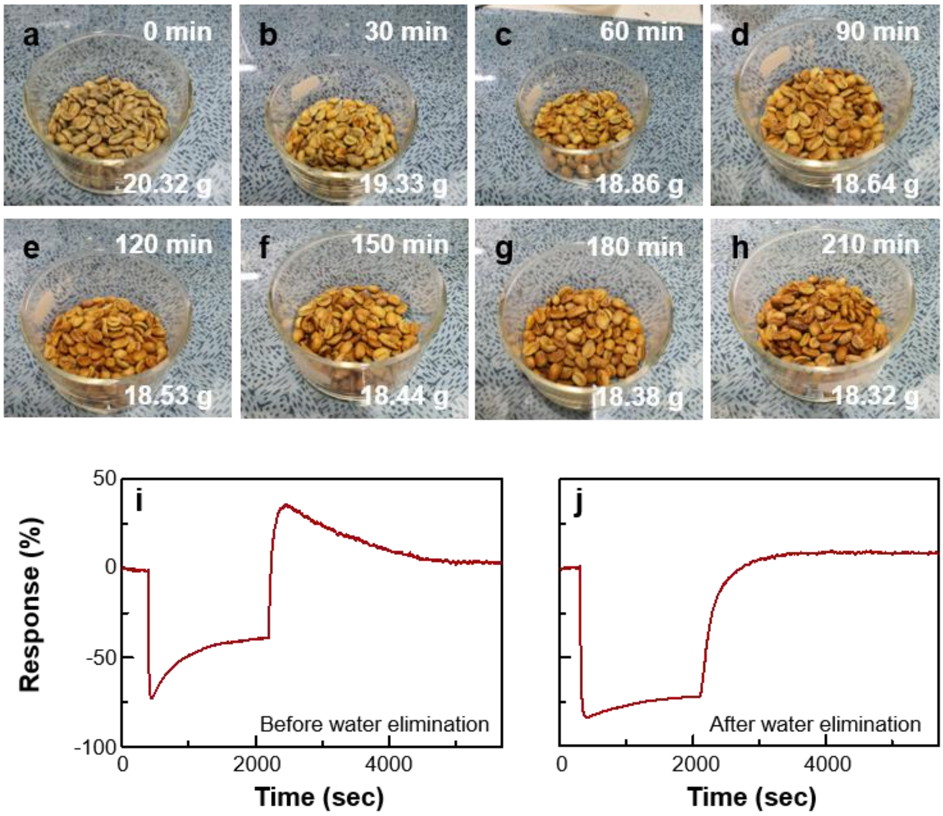

Figure S17. Photographs of heated coffee beans at 150 °C with altering time of (a) 0, (b) 30, (c) 60, (d) 90, (e) 120, (f) 150, (g) 180, and (h) 210 min. Gas response of pristine ZnO (i) before and (j) after elimination of moisture in the coffee beans at 250 °C, respectively.

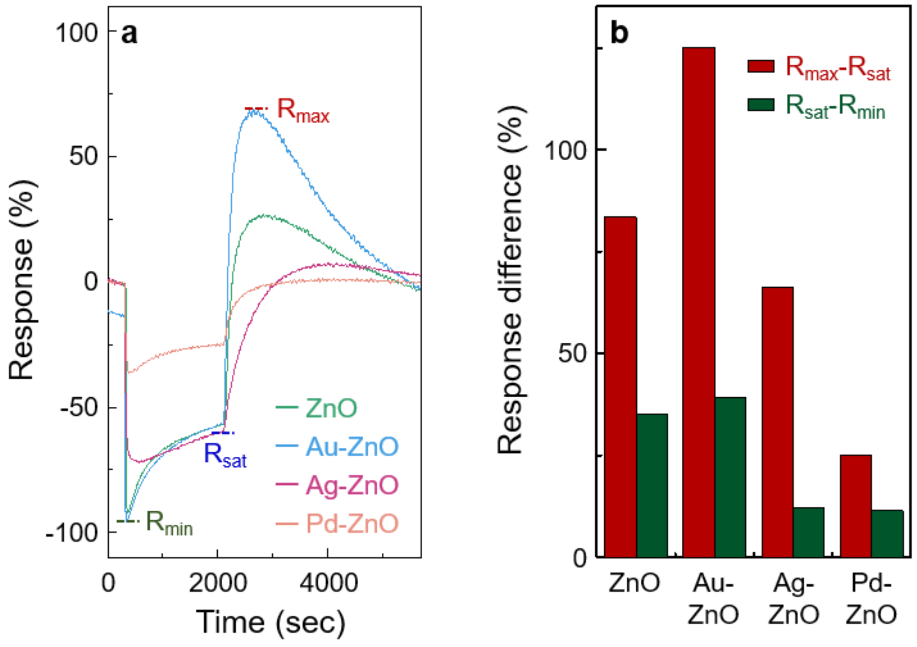

Figure S18. (a) Extracted gas response of pristine ZnO and metal NPs hybridized ZnO for Ethiopia coffee beans at 250 ^{o}C. (b) Representative constant (R_{max} - R_{sat}, R_{sat} - R_{min}) for over shooting effect extracted from gas response of pristine and metal NPs hybridized ZnO for Ethiopia coffee beans.

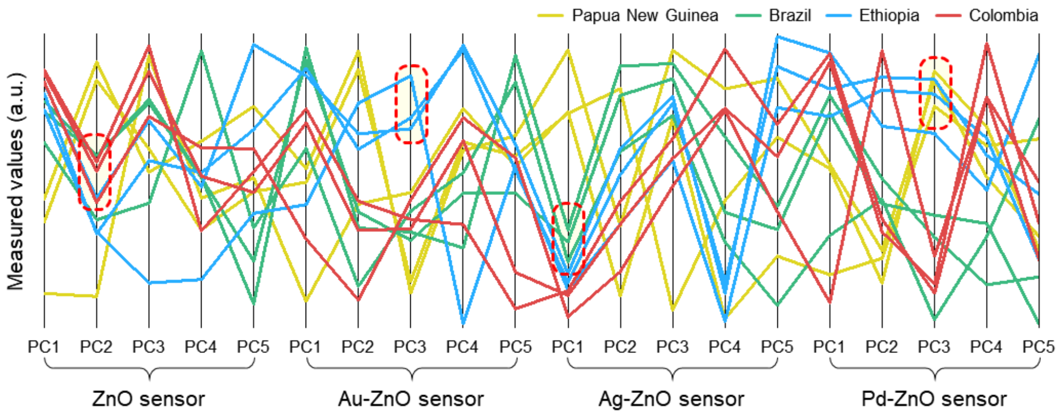

Figure S19. Parallel coordinate plot of five-representative PC scores for each ZnO-based artificial nose (pristine ZnO, Au NPs-ZnO, Ag NPs-ZnO, and Pd NPs-ZnO based sensors) from exponential fitting parameters for the coffee vapor response curves under response, saturation, and recovery stages.

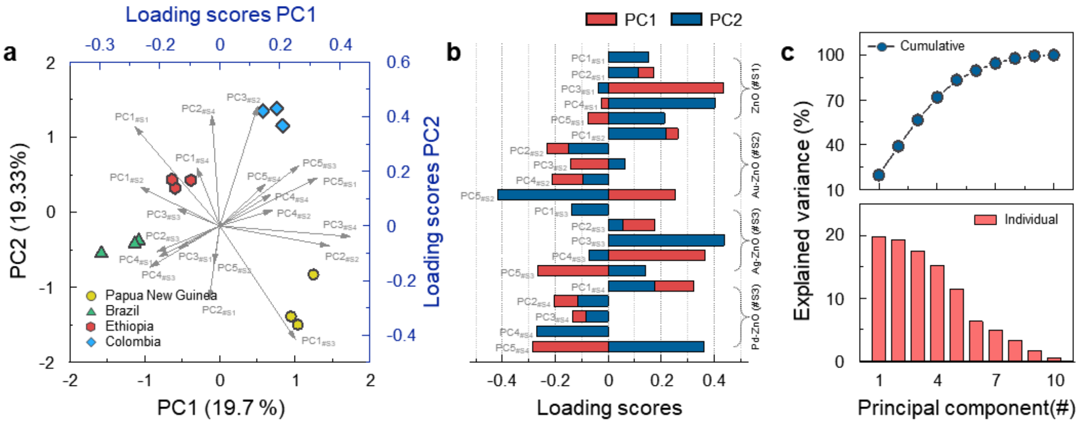

Figure S20. Double-PCA results. (a) PCA scatter plot for four-different types of coffee vapors. (b) PCA Loading score of two-representative PC components (PC1 and PC2) for the 20-initial PC scores (five PC scores (PC1, PC2, …, and PC5) for 4-different types of ZnO-based sensor). (c) Explained variance plot by PCs (top: cumulative, bottom: individual).

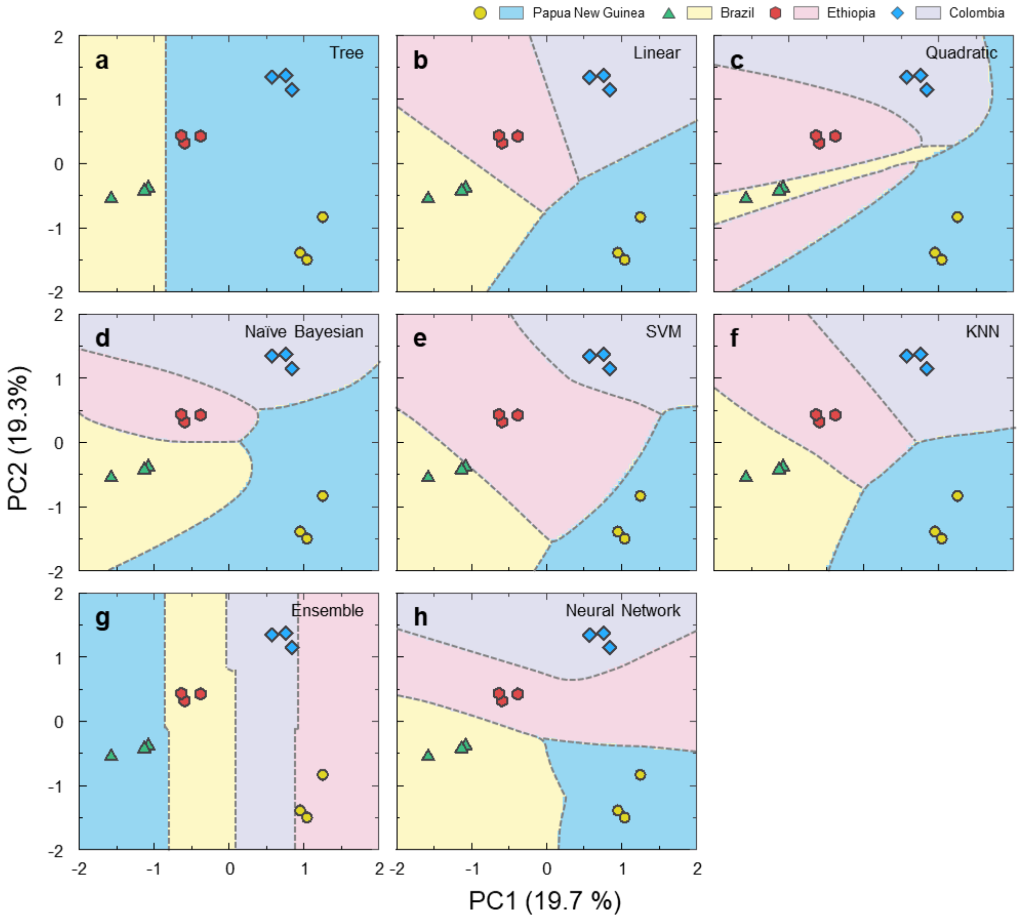

Figure S21. Classification boundary map of four-different coffee vapors with varied training model with double-PCA-assisted data manipulation for (a) decision tree model, (b) linear discriminant model, (c) quadratic discriminant model, (d) Naïve Bayes model, (e) support vector machine, (f) k-nearest neighbor, (g) ensemble bagged trees, and (h) neural networks.

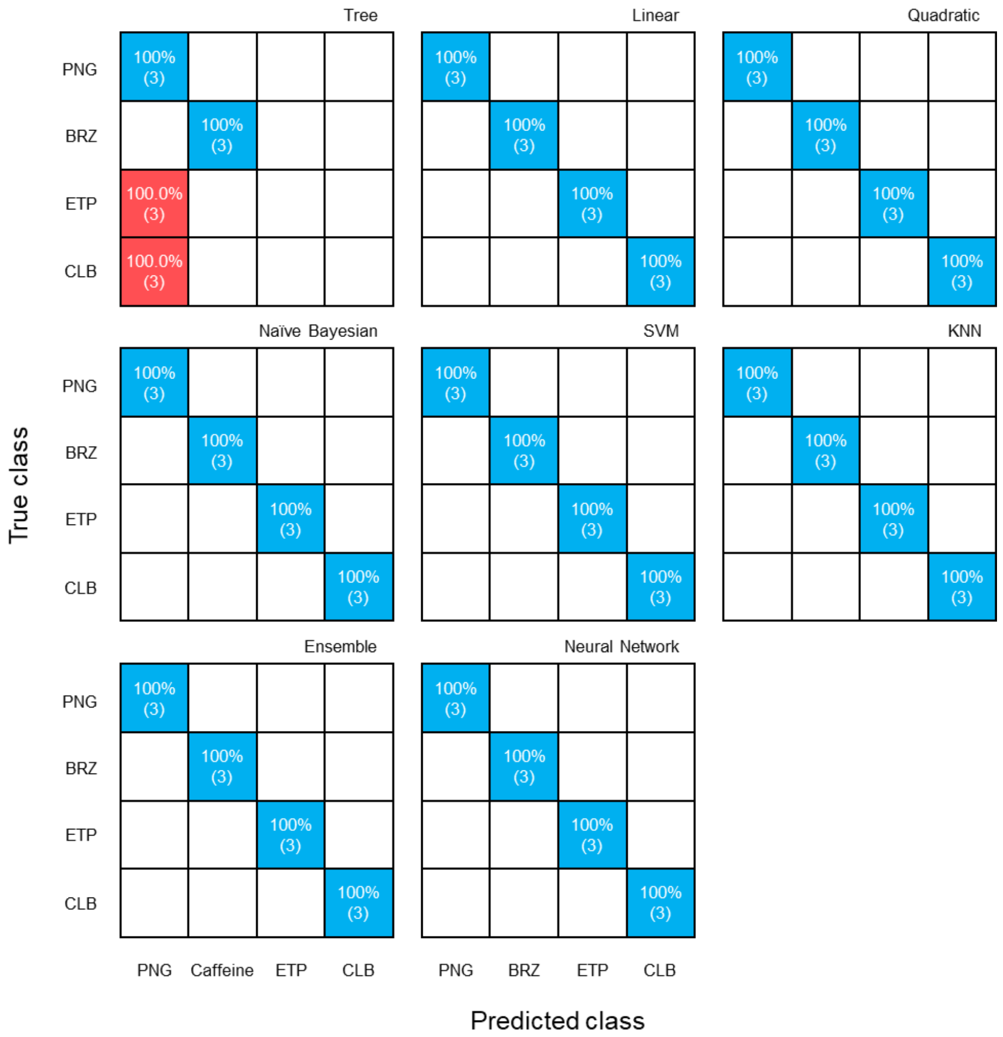

Figure S22. Confusion matrix of varied classifiers using double-PCA data manipulation such as decision tree model, linear discriminant model, quadratic discriminant model, Naïve Bayes model, support vector machine, k-nearest neighbor, ensemble bagged trees, and neural networks.

Supplementary Note 3.

Except for the tree classifier, overall models show 100% of prediction accuracy. It is likely due to a simplified algorithm for determining decision boundaries, which can be further optimized for better performance.

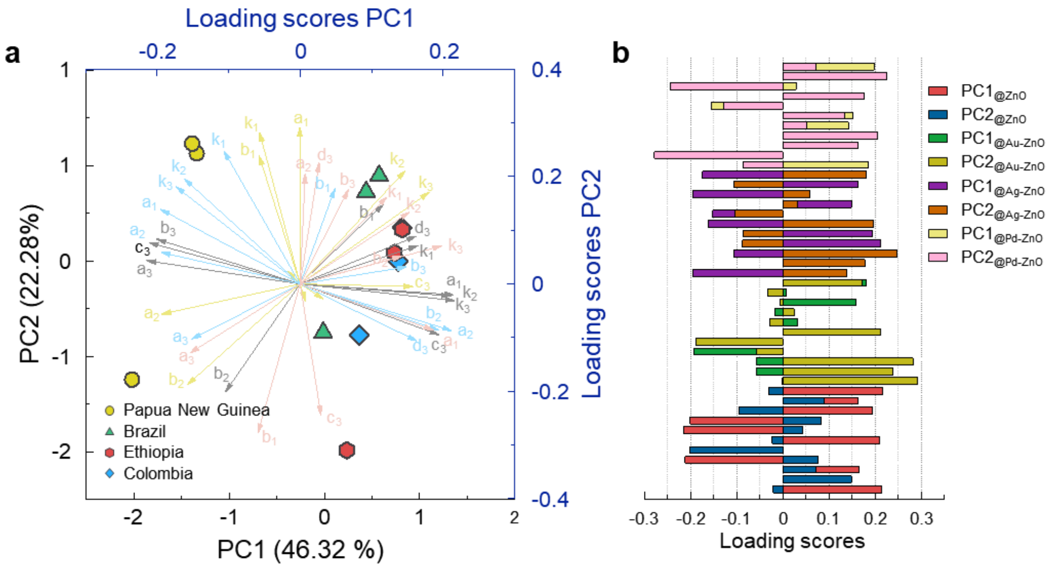

Figure S23. Single PCA results. (a) PCA scatter plot for four-different coffee vapors with initial PC scores from each ZnO-based sensor. (b) Loading scores of PC1 and PC2 for each 11-extracted exponential fitting parameters of ZnO-based sensor array.

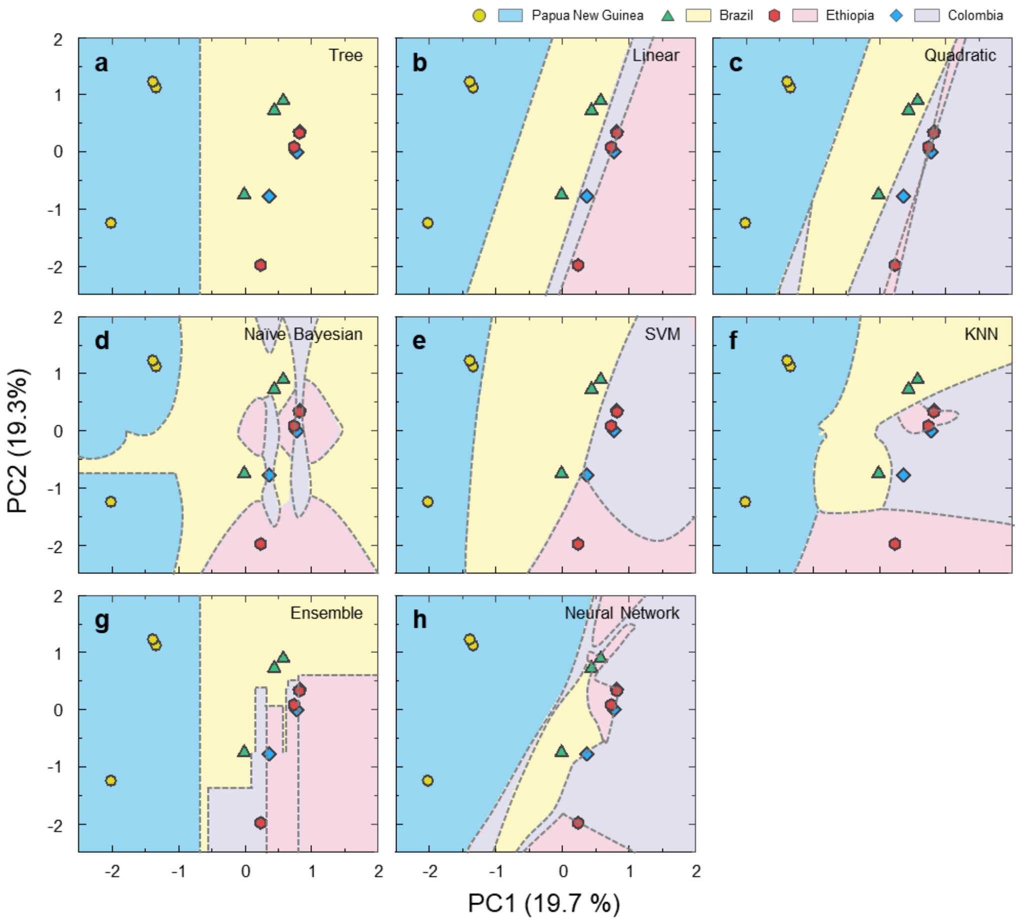

Figure S24. ML results using initial-PCA feature data. Classification boundary map of four-different coffee vapors with varied training model with initial-PCA-assisted data manipulation for (a) decision tree model, (b) linear discriminant model, (c) quadratic discriminant model, (d) Naïve Bayes model, (e) support vector machine, (f) k-nearest neighbor, (g) ensemble bagged trees, and (h) neural networks.

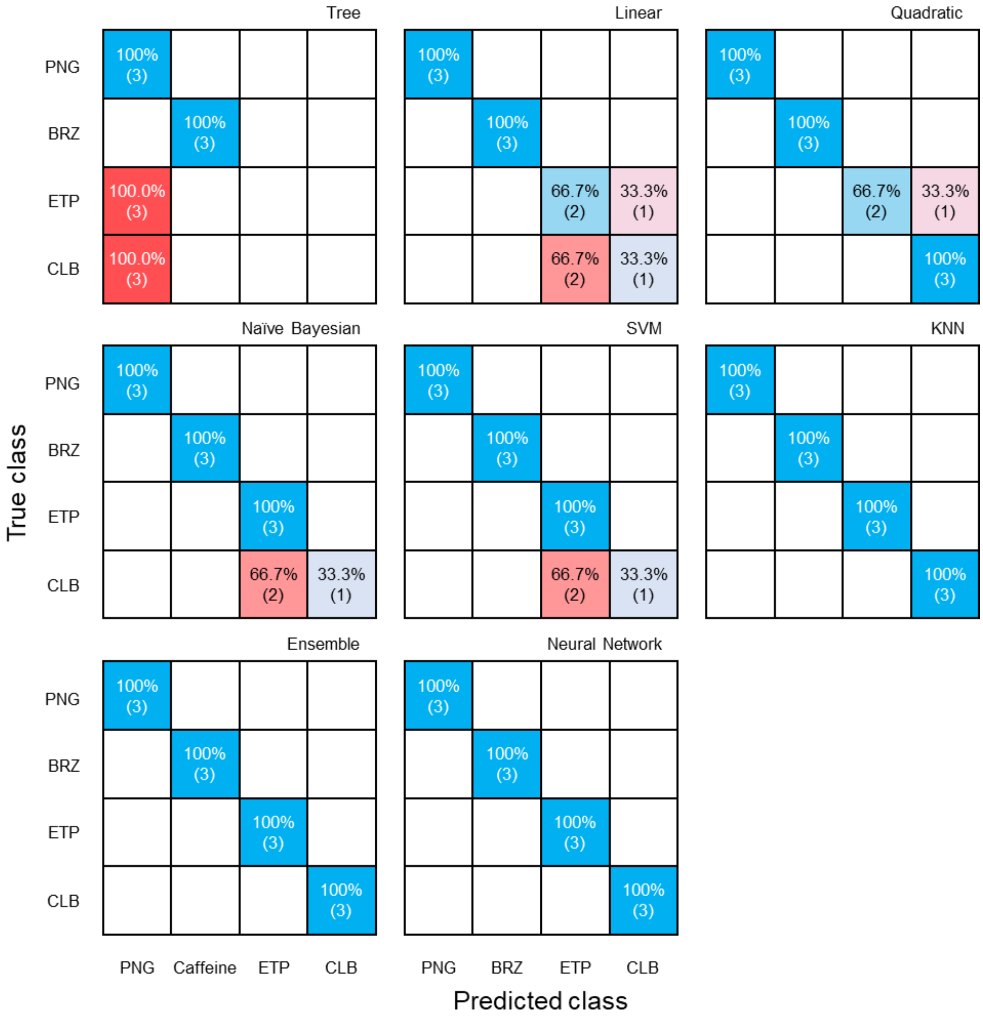

Figure S25. Confusion matrix of varied classifiers using initial-PCA data manipulation such as decision tree model, linear discriminant model, quadratic discriminant model, Naïve Bayes model, support vector machine, k-nearest neighbor, ensemble bagged trees, and neural networks.

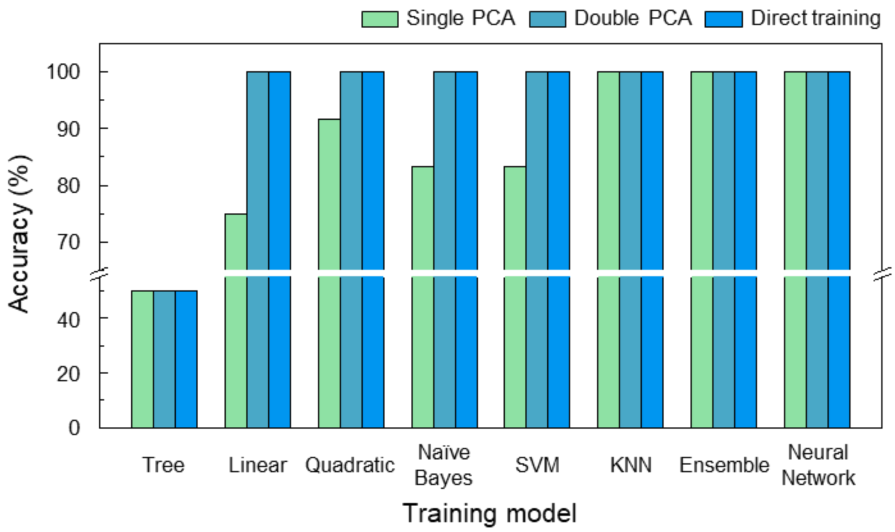

Figure S26. Summarized prediction accuracy of varied training model to compare with data manipulation process by initial PCA, double PCA, and direct input.

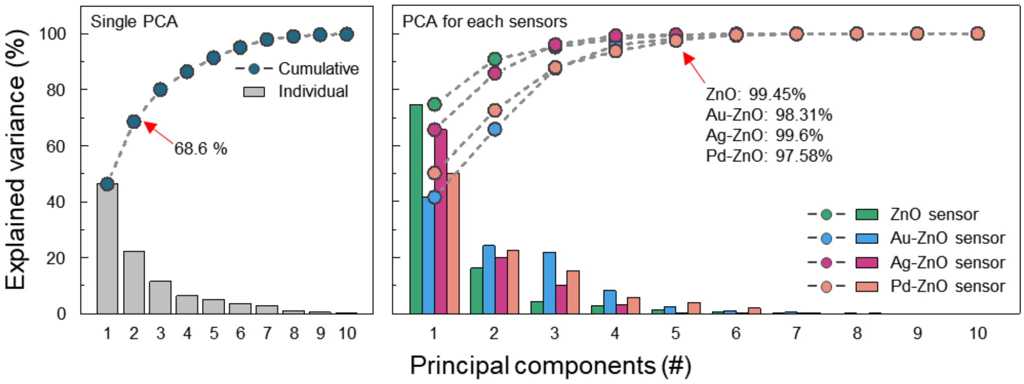

Figure S27. Comparison of the explained variance plot (left) for single-PCA process from 44-extracted fitting parameters and (right) first order PCA for each ZnO-based sensor from 11 extracted fitting parameters.

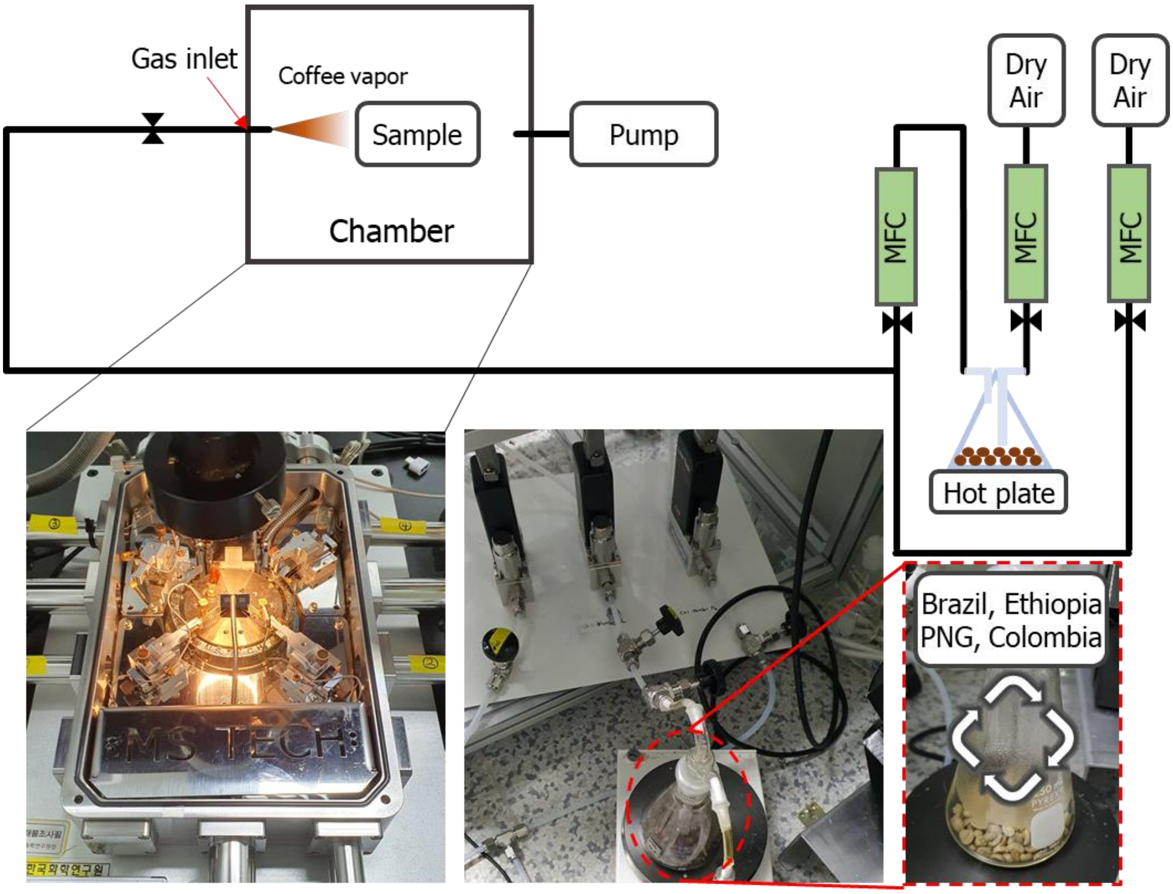

Figure S28. Schematic diagram of the coffee aroma measurement system, including the gas flow setup for bubbling coffee aroma by introducing dry air through the Erlenmeyer flask.
